# *Streptococcus pneumoniae* hijacks host autophagy by deploying CbpC as a decoy for Atg14 depletion

**DOI:** 10.15252/embr.201949232

**Published:** 2020-04-02

**Authors:** Sayaka Shizukuishi, Michinaga Ogawa, Satoko Matsunaga, Mikado Tomokiyo, Tadayoshi Ikebe, Shinya Fushinobu, Akihide Ryo, Makoto Ohnishi

**Affiliations:** ^1^ Department of Bacteriology I National Institute of Infectious Diseases Tokyo Japan; ^2^ Department of Microbiology Yokohama City University Graduate School of Medicine Kanagawa Japan; ^3^ School of Veterinary Medicine Azabu University Kanagawa Japan; ^4^ Department of Biotechnology Graduate School of Agricultural and Life Sciences The University of Tokyo Tokyo Japan; ^5^ Collaborative Research Institute for Innovative Microbiology The University of Tokyo Tokyo Japan

**Keywords:** Atg14, autophagy, CbpC, p62, *Streptococcus pneumoniae*, Autophagy & Cell Death, Microbiology, Virology & Host Pathogen Interaction, Signal Transduction

## Abstract

Pneumococcal cell surface‐exposed choline‐binding proteins (CBPs) play pivotal roles in multiple infectious processes with pneumococci. Intracellular pneumococci can be recognized at multiple steps during bactericidal autophagy. However, whether CBPs are involved in pneumococci‐induced autophagic processes remains unknown. In this study, we demonstrate that CbpC from *S. pneumoniae* strain TIGR4 activates autophagy through an interaction with Atg14. However, *S. pneumoniae* also interferes with autophagy by deploying CbpC as a decoy to cause autophagic degradation of Atg14 through an interaction with p62/SQSTM1. Thus, *S. pneumoniae* suppresses the autophagic degradation of intracellular pneumococci and survives within cells. Domain analysis reveals that the coiled‐coil domain of Atg14 and residue Y83 of the dp3 domain in the N‐terminal region of CbpC are crucial for both the CbpC–Atg14 interaction and the subsequent autophagic degradation of Atg14. Although homology modeling indicates that CbpC orthologs have similar structures in the dp3 domain, autophagy induction through Atg14 binding is an intrinsic property of CbpC_._ Our data provide novel insights into the evolutionary hijacking of host‐defense systems by intracellular pneumococci.

## Graphical abstract

Pneumococcal cell surface‐exposed choline‐binding proteins play pivotal roles in multiple infectious processes. CbpC promotes selective autophagy of Atg14 through an interaction with p62, thereby dampening degradation and xenophagy of *S. pneumonia*.

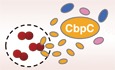

## Introduction

*Streptococcus pneumoniae* is a major, encapsulated gram‐positive pathogen that causes diseases including community‐acquired pneumonia, meningitis, and sepsis 1, 2. During severe infections, *S. pneumoniae* colonization of nasopharyngeal epithelial cells can lead to epithelial barrier penetration and entrance into the bloodstream and brain via the blood–brain barrier 1, 2. Although multivalent pneumococcal polysaccharides and conjugate vaccines are available and generally effective, they also have major shortcomings with respect to the emergence of vaccine‐resistant serotypes (serotype replacement) 2, 3. The increasing prevalence of antibiotic‐resistant pneumococci is a global problem 3. Therefore, alternative therapeutic approaches are needed. However, progress has been limited by an incomplete understanding of virulence factors and the intracellular fate of *S. pneumoniae*.

**Figure 1:**
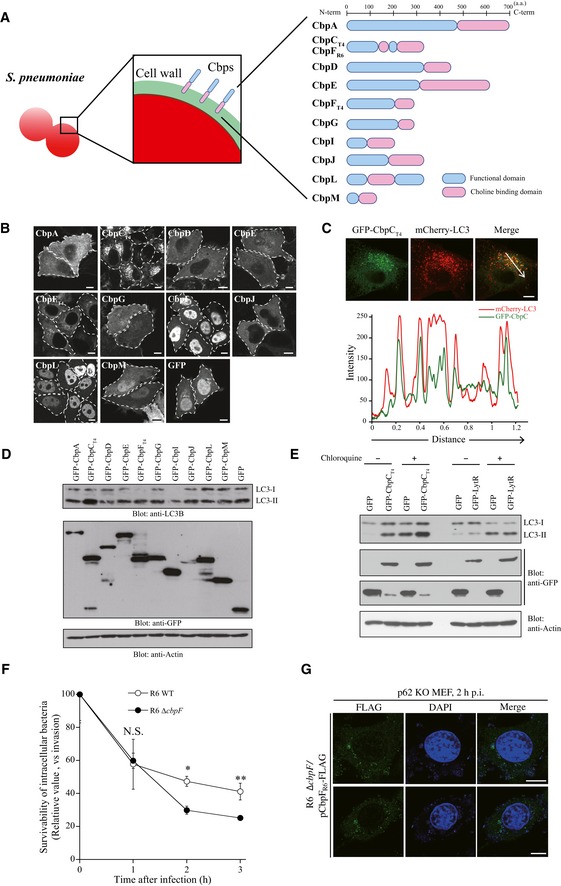
The pneumococcal CbpC protein can activate autophagy, but can also facilitate intracellular pneumococcal survival APneumococcal CBPs used in (B).BConfocal images of HeLa cells transiently expressing GFP‐CBPs or GFP. The dotted lines show each cell shape. Scale bars, 10 μm.CConfocal images of HeLa cells transiently expressing GFP‐CbpC_T4_ and mCherry‐LC3 (upper). The fluorescence intensities of GFP‐CbpC_T4_ (green) and mCherry‐LC3 (red) along the arrow are shown in the graph at the bottom.DLysates from 293T cells transiently expressing GFP‐Cbps or GFP were subjected to SDS–PAGE and analyzed by immunoblotting using antibodies against LC3, GFP, or actin.ELysates from 293T cells transiently expressing GFP‐CbpC_T4_, GFP‐LytR, or GFP in the presence or absence of chloroquine were subjected to SDS–PAGE and analyzed by immunoblotting using antibodies against LC3, GFP, or actin.FMEFs were infected with *S. pneumoniae* R6 WT or Δ*cbpF* for the indicated periods, and the intracellular survival of bacteria expressed as the number of CFUs.Gp62‐KO MEF cells infected with *S. pneumoniae* R6 Δ*cbpF*/pCbpF_R6_‐FLAG for 2 h were fixed and stained with DAPI and an anti‐FLAG antibody, and representative epifluorescence images are shown. Scale bars, 10 μm.Data information: In (F), data represent mean ± SEM of 3 biological replicates. Student's *t*‐test was used to calculate statistical significance. *P < 0.05, **P < 0.01.Source data are available online for this figure.

Pneumococcal cell surface proteins, including LPXTG motif‐containing proteins, lipoproteins, and choline‐binding proteins (referred to as CBPs or Cbps), are key pathogenic factors 4, 5, 6, 7, 8 and are viewed as primary therapeutic targets 7. *S. pneumoniae* has an absolute nutritional requirement for choline. Its characteristic cell wall is composed of lipoteichoic and teichoic acids and is decorated with phosphocholine (PCho) 4, 7. *S. pneumoniae* have more than 15 CBPs, with PCho acting as a scaffold for all of them at the cell wall (Fig 1A) 4, 7. All CBP family proteins share choline‐binding modules (CBMs) composed of choline‐binding repeats (CBRs), which facilitate their binding to the cell wall 4, 7. To date, the crystal structures of 7 pneumococcal CBM‐containing proteins have been solved, including CbpE 9, CbpF 10, CbpJ 11, CbpL 12, and LytA 13. Although it has been reported that CBPs are involved in pathogenic functions of pneumococci, including adhesion to host cells, bacterial autolysis, and complement activation, the functional understanding of CBPs is incomplete 4, 5, 6, 7, 8.

Xenophagy functions as an innate host‐defense system against microbial intruders, providing a first line of defense 14, 15, 16. Upon intracellular pathogen invasion, xenophagy is activated in host cells by the recognition of bacterial components or infection processes via multiple cytosolic sensors 14. However, many intracellular bacterial pathogens have evolved strategies to subvert xenophagy, including evading autophagic recognition, dampening autophagosome formation, and manipulating autophagosome–lysosome fusion 14, 17, 18, 19. Previously, we showed that intracellular *S. pneumoniae* can be recognized by bactericidal autophagy 20; however, whether and how these autophagic processes are manipulated by pneumococcal virulence factors are mostly unknown.

In this study, we demonstrated that CbpC from *S. pneumoniae* strain TIGR4 induces autophagy by interacting with Atg14. Our data also revealed that the p62–CbpC–Atg14 complex causes the selective autophagy targeting Atg14, which eventually attenuates the autophagic degradation of intracellular pneumococci.

## Results

### The pneumococcal CbpC protein can activate autophagy and also facilitate intracellular pneumococcal survival

**Figure EV1:**
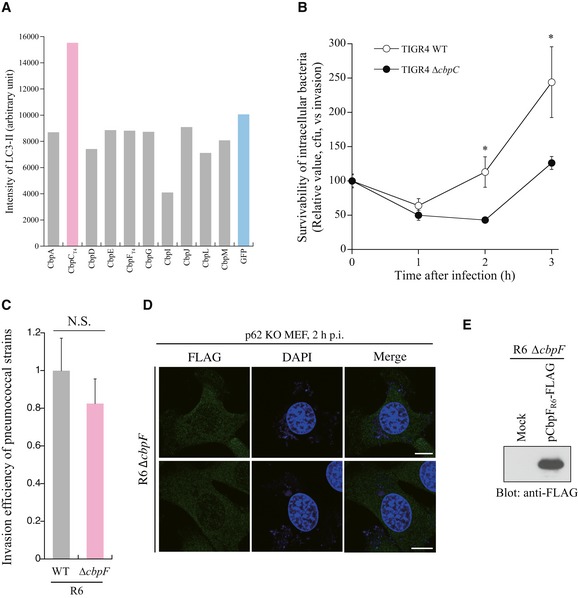
Pneumococcal CbpC protein can act not only as an autophagy activator but rather as a facilitator for intracellular pneumococcal survivability AQuantification of the band intensities shown in Figure 1D.BMEFs were infected with *S. pneumoniae* TIGR4 WT or Δ*cbpC* for the indicated periods, and intracellular survivability of bacteria was determined as CFU (colony‐forming units, *n* = 3).CMEFs were infected with *S. pneumoniae* R6 WT or Δ*cbpC,* and invasion efficiency of bacteria was determined by CFU.Dp62‐KO MEF cells infected with *S. pneumoniae* R6 *ΔcbpF* for 2 h were fixed and stained with DAPI and an anti‐FLAG antibody. Representative epifluorescence images are shown. Scale bars, 10 μm.ELysates from *S. pneumoniae* R6 Δ*cbpF* or Δ*cbpF* expressing CbpF_R6_‐FLAG were subjected to SDS–PAGE and analyzed by immunoblotting using an anti‐FLAG antibody.Data information: In (B, C), data represent mean ± SEM of 3 biological replicates. Student's *t*‐test was used to calculate statistical significance. *P < 0.01. N.S., not significant.Source data are available online for this figure.

*Streptococcus pneumoniae* undergoes spontaneous autolysis during infection, during which pneumococcal cell wall‐associated components, including CBPs and LPXTG proteins, diffuse into the cytosol of host cells through endosomal membrane pores formed by pneumolysin, a cholesterol‐binding cytolysin 21. Therefore, we investigated the intracellular functions of CBPs in pneumococcal virulence (Fig 1A). In this study, we used the nomenclature from the *S. pneumoniae* TIGR4 strain. When Cbps such as CbpA, C, D, E, F, G, I, J, L, and M were ectopically expressed in HeLa cells as green fluorescence protein (GFP)‐fusion proteins, we found that GFP‐CbpC (CbpC from TIGR4, hereafter referred as CbpC_T4_) caused the formation of intracellular inclusion bodies, reminiscent of autophagic puncta (Fig 1B). CbpC_T4_ is one of the most abundant proteins in the pneumococcal cell wall 10, 22. Upon transient expression in HeLa cells, GFP‐CbpC_T4_ co‐localized with mCherry‐LC3, an intrinsic autophagosome marker 23 (Fig 1C). The amount of LC3‐II (a membrane‐bound form of LC3) exclusively increased in cells expressing GFP‐CbpC_T4_ (Figs 1D and EV1A). To determine whether the increase in LC3‐II and GFP‐LC3‐positive puncta was caused by autophagy activation or inhibited autophagic degradation, we performed autophagic flux assays in chloroquine‐treated, GFP‐CbpC_T4_‐expressing cells. Upon chloroquine treatment, GFP‐CbpC_T4_‐induced LC3‐II accumulation was further augmented, indicating that transiently expressed CbpC_T4_ was involved in activating autophagy (Fig 1E).

**Figure EV2:**
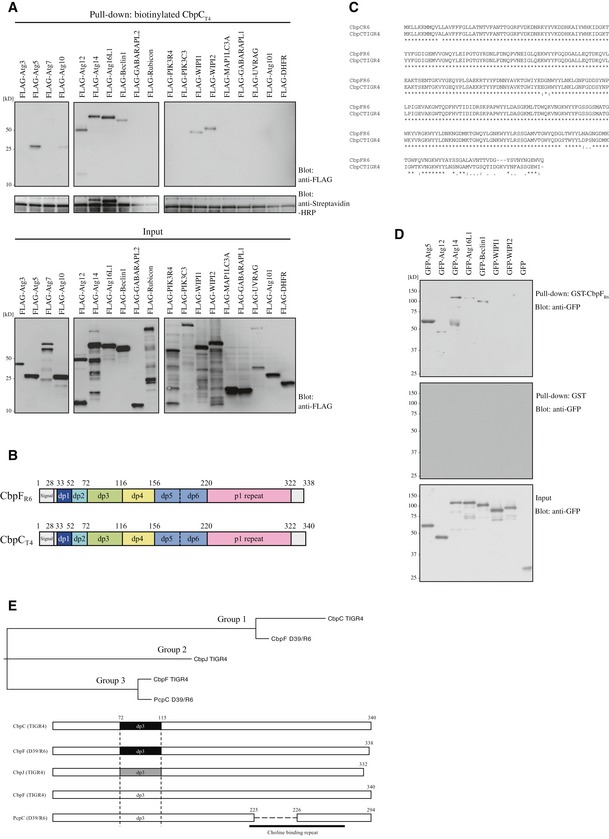
Screening of autophagy‐related proteins that interacted with CbpC and sequence analysis of CbpC homologs in the genomes of the *S. pneumoniae* TIGR4 and D39/R6 strains AStreptavidin‐pulldown assays using *in vitro*‐translated biotinylated CbpC_T4_ and FLAG‐tagged autophagy‐related proteins. Bound proteins were analyzed by immunoblotting using an anti‐FLAG antibody.BDiagram of CbpF_R6_ and CbpC_T4_.CSequence alignment of CbpF_R6_ and CbpC_T4_. The identical amino acid residues are indicated by asterisk, and conservative changes are shown by dots.DGST‐pulldown assays using GST‐CbpF_R6_ or GST and lysates from 293T expressing the indicated GFP‐fused proteins or GFP. Bound proteins were analyzed by immunoblotting using an anti‐GFP antibody.EPhylogenetic tree and schematic representations of CbpC homologs. Source data are available online for this figure.

To investigate the physiological role of CbpC in *S. pneumoniae* infection, we conducted intracellular‐survivability assays using *S. pneumoniae* R6 or TIGR4. CbpF_R6_ (CbpF from R6, hereafter referred as CbpF_R6_) is a CbpC_T4_ ortholog with 97% sequence similarity (312/340 amino acids) in the N‐terminal functional region (Fig EV2B and C) 10. Upon infection with the wild‐type (WT) and ∆*cbpF*_*R6*_ or ∆*cbpC*_*T4*_ strains derived from *S. pneumoniae* R6 or TIGR4, we found that the survival of ∆*cbpF*_*R6*_ or ∆*cbpC*_*T4*_ strains was significantly lower than that of WT bacteria at the later stage of infection, whereas CbpF_R6_ and CbpC_T4_ had no adverse effect on bacterial invasion into host cells (Figs 1F and EV1B and C). Next, we examined whether bacterial autolysis releases free CbpC into the cytosol. Upon infection with *S. pneumoniae* R6 ∆*cbpF*/pCbpF_R6_‐FLAG, CbpF_R6_ signals were detected proximal to intracellular pneumococci, but also free in the cytosol, whereas these signals were completely abolished in ∆*cbpF*‐infected cells (Figs 1G and EV1D and E).

### Identification of Atg14 as a CbpC‐interacting protein

**Figure 2:**
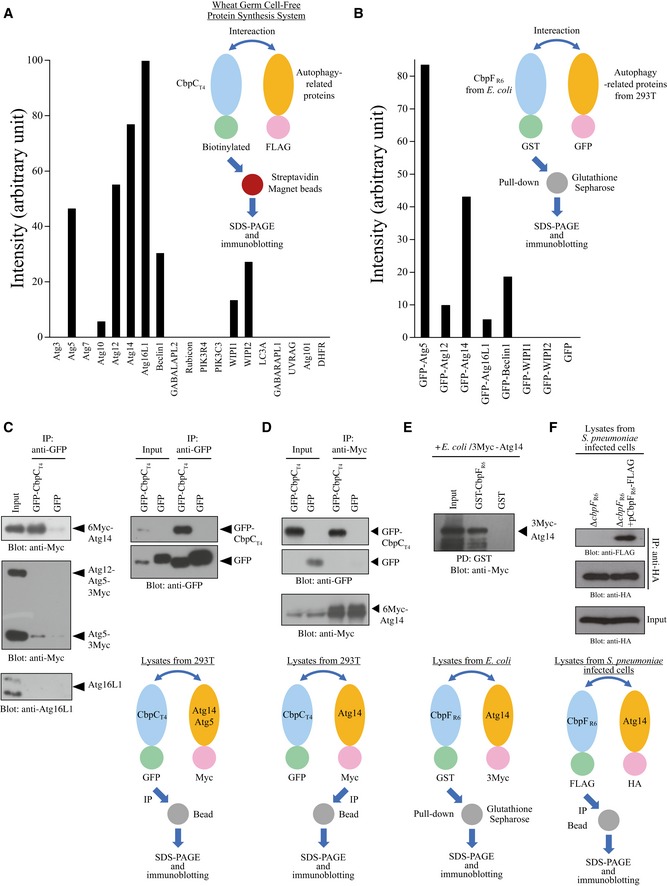
Identification of Atg14 as a CbpC‐interacting protein AQuantification of the band intensities following streptavidin‐based pulldown assays using the *in vitro‐*translated biotinylated CbpC_T4_ and FLAG‐tagged autophagy‐related proteins shown in Fig EV2A.BQuantification of the band intensities following GST‐pulldown assays with 293T cell lysates expressing the autophagy‐related proteins and recombinant GST‐CbpF_R6_ shown in Fig EV2D.CLysates from 293T cells transiently expressing 3Myc‐Atg14 or Atg5‐3Myc, and GFP‐CbpC_T4_ or GFP were immunoprecipitated with GFP‐Trap, and the bound proteins were analyzed by immunoblotting.DLysates from 293T cells transiently expressing GFP‐CbpC_T4_ or GFP, and 6Myc‐Atg14 were immunoprecipitated with an anti‐Myc antibody and Protein G PLUS‐Agarose. The bound proteins were analyzed by immunoblotting.ERecombinant GST‐CbpF_R6_ or GST, and 3Myc‐Atg14 were used for GST‐pulldown assays. The bound proteins were analyzed by immunoblotting using an anti‐Myc antibody.FMEFs infected with R6 Δ*cbpF* expressing CbpF_R6_‐FLAG for 2 h were subjected to IP and assayed using anti‐HA agarose beads. The bound proteins were analyzed by immunoblotting using an anti‐FLAG antibody. Schematic representations indicating assay designs in (C–F) are shown below the blots. Source data are available online for this figure.

We next investigated the mechanism whereby CbpC_T4_ induces autophagy. We hypothesized that CbpC_T4_ could functionally interact with autophagy‐related proteins. Therefore, we conducted comprehensive protein–protein interaction analysis. As a first screening, we synthesized a series of autophagy‐related proteins using a wheat cell‐free system and then performed pulldown assays. Our data demonstrated that CbpC_T4_ could interact with seven autophagy‐related proteins, including Atg5, 12, 14, 16L1, Beclin1, WIP1, and WIPI2 (Figs 2A and EV2A). To exclude candidates with non‐specific binding, we performed a second screening by performing glutathione S transferase (GST)‐based pulldown assays using 293T cell lysates expressing GFP‐fusion proteins with Atg5, 12, 14, 16L1, Beclin1, WIPI1, or WIPI2. As GST‐CbpC_T4_ expression inhibited *Escherichia coli* growth, we prepared a plasmid encoding GST‐CbpF_R6_ for expression in *E. coli* 10. Based on the results of the second screen, we focused on Atg5 and Atg14 as CbpC_T4_‐interacting candidates (Figs 2B and EV2D). Further immunoprecipitation (IP) assays in 293T cells revealed the specific interaction of CbpC_T4_ with Atg14 (Fig 2C and D). Furthermore, GST‐pulldown assays using recombinant GST‐CbpF_R6_ and 3Myc‐Atg14 corroborated the direct interaction of CbpF_R6_ with Atg14 (Fig 2E). Finally, we confirmed the CbpC–Atg14 interaction in *S. pneumoniae*‐infected cells. When MEFs (mouse embryonic fibroblast cells) stably expressing hemagglutinin (HA)‐tagged Atg14 (MEFs/HA‐Atg14) were infected with *S. pneumoniae* R6 ∆*cbpF*/pCbpF_R6_‐FLAG, a CbpF_R6_–FLAG–HA–Atg14 interaction was clearly detected during infection (Fig 2F).

### CbpC can act as a decoy for autophagic Atg14 degradation and xenophagy subversion

Atg14 plays two roles in autophagy; the first is as a component of the autophagy‐specific class III phosphatidylinositol 3‐kinase complex through Beclin1‐binding 24, 25, 26, and the second is as a regulator of autophagosome‐lysosome fusion through Stx17 binding 27, 28. Therefore, we hypothesized that *S. pneumoniae* could promote CbpC‐driven selective autophagy during early infection and that CbpC–Atg14 binding would lead to Atg14 degradation, which, in turn, would reduce autophagosome–lysosome fusion and bacterial degradation.

**Figure EV3:**
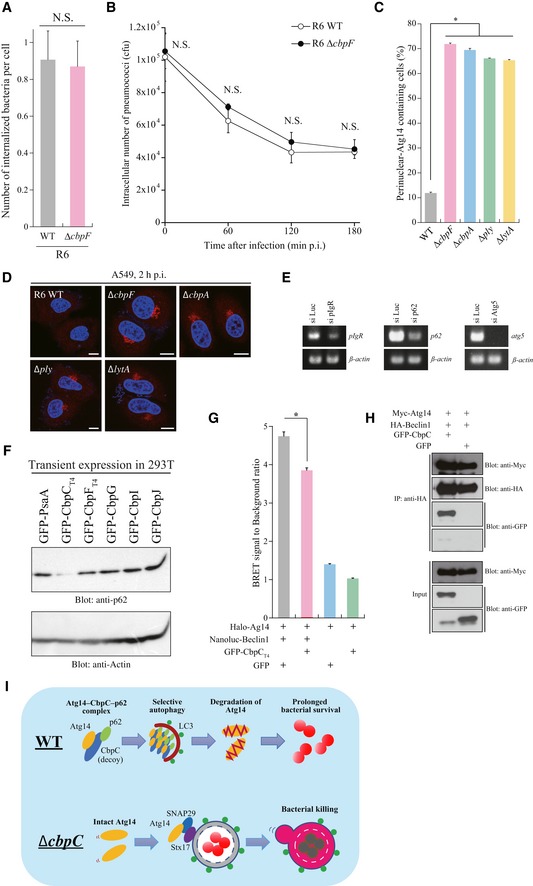
CbpC can bind to p62 and act as a decoy for autophagic degradation of Atg14 to suppress autophagic degradation AMEFs were infected with *S. pneumoniae* R6 WT or Δ*cbpF*, and the number of internalized bacteria per cell was determined.BAtg5‐KO MEFs were infected with *S. pneumoniae* R6 WT or Δ*cbpF* for the indicated periods, and the intracellular survival of bacteria was determined and expressed as CFUs.CA549 cells infected with the indicated *S. pneumoniae* strains for 2 h were fixed and stained with DAPI and an anti‐Atg14 antibody, and the percentages of perinuclear‐localizing Atg14 containing cells were quantified.DRepresentative epifluorescence images of the data presented in (C) are shown. Scale bars, 10 μmEKnockdown effects of the indicated siRNAs were evaluated by RT–PCR and visualized by agarose gel electrophoresis.FLysates from 293T cells transiently expressing the indicated proteins were subjected to SDS–PAGE and analyzed by immunoblotting using the indicated antibodies.GQuantification of NanoBRET signals in 293A cells transiently expressing Nanoluc‐Beclin1 and HaloTag‐Atg14 in the presence or absence of GFP‐CbpC or GFP.HLysates from 293T cells transiently expressing the indicated proteins were subjected to IP assays using anti‐HA beads, and bound proteins were analyzed by Western blotting using the indicated antibodies.ISchematic diagram of p62–CbpC–Atg14‐driven autophagy subversion in pneumococcal infection.Data information: In (A, B, C, G), data represent mean ± SEM of 3 biological replicates. Student's *t*‐test was used to calculate statistical significance. *P < 0.01, N.S., not significant.Source data are available online for this figure.

**Figure 3:**
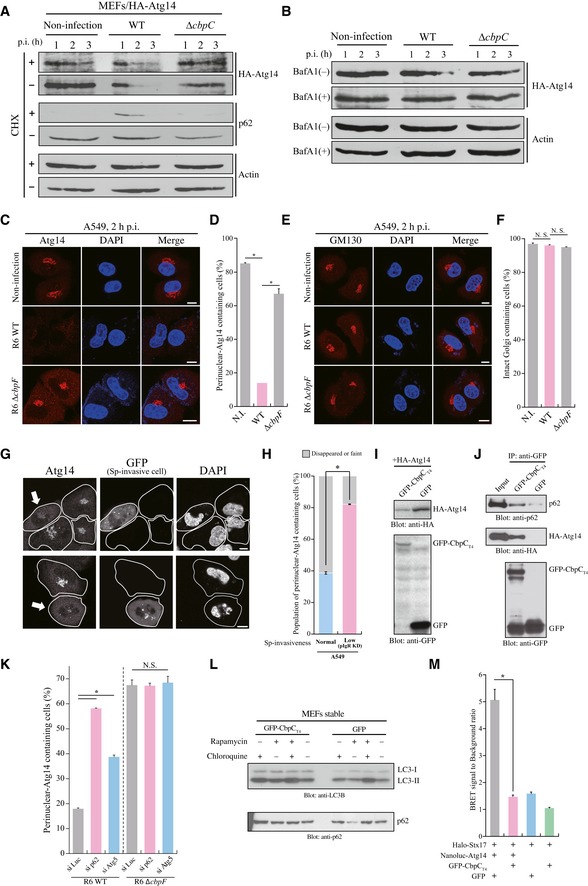
CbpC could bind to p62 and act as a decoy for autophagic degradation of Atg14 to suppress autophagic degradation ALysates from MEFs stably expressing HA‐Atg14 infected with *S. pneumoniae* TIGR4 WT or Δ*cbpC* for 1, 2, or 3 h in the presence or absence of cycloheximide were subjected to SDS–PAGE and analyzed by immunoblotting with the indicated antibodies.BMEFs/HA‐Atg14 cells were infected with *S. pneumoniae* TIGR4 WT or Δ*cbpC* for 1, 2, or 3 h in the presence or absence of Bafilomycin A1 (BafA1). The lysates were subjected to SDS–PAGE and analyzed by immunoblotting with the indicated antibodies.C–F(C, E) A549 cells were infected with *S. pneumoniae* R6 WT or Δ*cbpC* for 2 h and fixed and stained with DAPI and an anti‐Atg14 or anti‐GM130 antibody. Representative epifluorescence images are shown. Scale bars, 10 μm. (D, F) The percentages of perinuclear‐localizing Atg14 containing cells in (C) or intact Golgi apparatus‐containing cells in (E) were quantified.G
*Streptococcus pneumoniae* (Sp) invasion‐permissive A549 cells (marked with GFP) and Sp invasion‐non‐permissive (pIgR knocked down) A549 cells were co‐cultured at a 1:3 ratio. Atg14‐disappearance experiments were conducted, and representative epifluorescence images are shown. Scale bars, 10 μm. The lines show each cell shape, and the arrows show invasion‐permissive A549 cells marked with GFP.HThe percentages of perinuclear‐localizing Atg14 containing cells in (G) were quantified.ILysates from 293T cells transiently expressing GFP‐CbpC or GFP, and HA‐Atg14 were subjected to SDS–PAGE and analyzed by immunoblotting using antibodies against HA or GFP.JLysates from 293T cells transiently expressing GFP‐CbpC_T4_ or GFP were immunoprecipitated using GST‐GFP‐Nanobody. Additionally, beads were mixed with lysates from 293T cells transiently expressing p62‐3Myc and HA‐Atg14, and bound proteins were analyzed by immunoblotting.KA549 cells treated with the indicated siRNAs were infected with *S. pneumoniae* R6 WT or Δ*cbpF* for the indicated durations. The cells were fixed and stained with DAPI and an anti‐Atg14 antibody, and percentages of perinuclear‐localizing Atg14 containing cells were quantified.LLysates from MEFs stably expressing GFP‐CbpC_T4_ or GFP in the presence or absence of rapamycin or chloroquine were subjected to SDS–PAGE and immunoblotted using antibodies against LC3 or p62.MQuantification of NanoBRET signals in 293A cells transiently expressing HaloTag‐Stx17 and Nanoluc‐Atg14 in the presence or absence of GFP‐CbpC or GFP.Data information: In (D, F, H, K, M), data represent mean ± SEM of 3 biological replicates. Student's *t*‐test was used to calculate statistical significance. *P < 0.01, N.S., not significant.Source data are available online for this figure.

To test this hypothesis, we infected MEFs/HA‐Atg14 with *S. pneumoniae* TIGR4 WT or ∆*cbpC* in presence or absence of cycloheximide (CHX) for 1, 2, or 3 h, and then, we examined whether the amount of HA‐Atg14 decreased during early infection in a CbpC‐dependent manner. Approximately 1 bacterium was internalized per cell under our experimental conditions, suggesting that most cells were comparably invaded by bacteria (Fig EV3A). Importantly, our findings revealed that the amount of HA‐Atg14 dramatically decreased in cells infected with WT bacteria after 2 h of infection, but not in cells infected with ∆*cbpC* bacteria (Fig 3A). We next determined whether autophagic degradation was suppressed in a CbpC‐dependent manner by employing p62 as a substrate for autophagic degradation. p62 degradation in CHX‐treated cells was dramatically suppressed in cells infected with WT bacteria, while that in ∆*cbpC* bacteria‐infected cells and even in uninfected cells rapidly increased (Fig 3A). This result strengthened our hypothesis that CbpC‐driven selective autophagy can cause Atg14 degradation, which ultimately suppresses bactericidal autophagy. We then investigated whether a defect in autophagic degradation would prevent the Atg14 degradation induced by *S. pneumoniae* infection. Notably, upon treatment with Bafilomycin A1, a v‐ATPase inhibitor, Atg14 degradation in WT bacteria‐infected cells was dramatically suppressed, and the Atg14 level was fully restored to that observed in ∆*cbpC* bacteria‐infected cells (Fig 3B). To investigate the physiological role of autophagy on the survival of ∆*cbpC*, we conducted intracellular‐survivability assays using Atg5‐knockout (KO) MEFs, which are autophagy‐deficient, and found that ∆*cbpC* survival was comparable to that of WT bacteria (Fig EV3B). These results supported our notion that CbpC‐driven autophagy during early infection can cause Atg14 depletion and suppress subsequent bactericidal autophagy. Furthermore, we investigated whether CbpC of *S. pneumoniae* could affect the abundance or localization of endogenous Atg14 in human lung epithelial cells. Notably, *S. pneumoniae* infection dramatically lowered Golgi‐resident Atg14 without affecting the structural integrity of the Golgi in a CbpC‐dependent manner (Fig 3C–F). Next, we examined whether Atg14 degradation was due to CbpC release in infected cells. When A549 cells were infected with an invasion‐deficient mutant (∆*cbpA*), an endosomal damage‐deficient mutant (∆*ply*), or a bacterial autolysis‐dampened mutant (∆*lytA*), the disappearance of Atg14 dramatically decreased to a similar level of ∆*cbpF*_*R6*_ (Fig EV3C and D). These results suggest that bacterial invasion, endosomal damage, and bacterial autolysis play pivotal roles in Atg14 degradation induced by *S. pneumoniae* infection. To further confirm the importance of bacterial invasion for *S. pneumoniae*‐induced Atg14 degradation, we constructed a polyclonal *S. pneumoniae* invasion setting using a co‐culture system. *S. pneumoniae* invasion‐permissive A549 cells (marked with GFP) and *S. pneumoniae* invasion‐non‐permissive (pIgR knockdown) A549 cells were co‐cultured at a 1:3 ratio, and *S. pneumoniae*‐induced Atg14 degradation was measured. Robust Atg14 degradation occurred in *S. pneumoniae* invasion‐permissive A549 cells (marked with GFP), but not in *S. pneumoniae* invasion‐non‐permissive (pIgR knockdown) A549 cells, clearly showing that bacterial invasion is essential for Atg14 degradation (Figs 3G and H, and EV3E). Taken together, these results support the idea that *S. pneumoniae* can manipulate autophagy by employing CbpC as a decoy to cause autophagic degradation of Atg14.

We then studied the Atg14‐degrading effect of CbpC_T4_ using 293T cells transiently expressing GFP‐CbpC_T4_ and HA‐Atg14, and we discovered that the amount of HA‐Atg14 dramatically decreased by co‐expressing GFP‐CbpC_T4_ (Fig 3I). To tackle the mechanism of CbpC_T4_‐induced Atg14 degradation through selective autophagy, we conducted IP assays using cells co‐expressing GFP‐CbpC_T4_, p62‐3Myc, and HA‐Atg14 and found that Atg14–CbpC–p62 complexes could form within cells (Fig 3J). Next, we examined whether Atg5 or p62 knockdown could suppress Atg14 degradation in *S. pneumoniae*‐infected A549 cells, and we found that the disappearance of perinuclear Atg14 caused by *S. pneumoniae* infection was dramatically suppressed by p62 knockdown. We also noticed that suppressive effect of Atg5 knockdown on the disappearance of perinuclear Atg14 was not as strong as that observed in p62‐knocked down cells, implying the partial involvement of an Atg5‐independent degradative pathway, such as the ubiquitin–proteasome system (Figs 3K and EV3E). These results suggested that selective autophagy through the Atg14–CbpC–p62 axis during early infection is involved in subsequent Atg14 degradation.

At a later stage of the autophagic process, Atg14 regulates autophagosome–lysosome fusion through Stx17 binding 27, 28. Therefore, we studied the autophagic degradation‐suppressive effect of CbpC in 293T cells, employing p62 as a reporter for autophagic flux. When CbpC was transiently expressed in 293T cells, acute autophagy induction via Atg14–CbpC–p62 signaling led to p62 degradation (Fig EV3F). Therefore, we constructed MEF cells stably expressing GFP‐CbpC_T4_ or GFP to determine the autophagic degradation‐suppressive effect of CbpC. Upon autophagy activation induced by rapamycin treatment, p62 was degraded in control MEFs stably expressing GFP (Fig 3L). In contrast, p62 degradation following rapamycin treatment was dramatically suppressed in MEFs stably expressing GFP‐CbpC_T4_, which also supported our notion that CbpC inhibits autophagosome–lysosome fusion and subsequent autophagic degradation. We therefore addressed whether CbpC‐induced Atg14 depletion could manipulate Atg14–Stx17 interactions. Thus, we measured direct interactions between these proteins in the presence or absence of CbpC in living cells using nano‐bioluminescence resonance energy transfer (NanoBRET) 29. Notably, the Atg14–Stx17 interaction was robustly suppressed in the presence of CbpC (Fig 3M); however, only slight inhibition of the Atg14–Beclin1 interaction occurred and CbpC–Atg14–Beclin1 complex formation was observed (Fig EV3G and H). Together, these results supported our notion that *S. pneumoniae* can manipulate autophagy by employing CbpC as a decoy to cause autophagic degradation of Atg14 and subsequent suppression of autophagosome–lysosome fusion and bactericidal autophagic degradation (Fig EV3I).

### Interaction of the dp3 loop of CbpC with the coiled‐coil domain (CCD) of Atg14

**Figure 4:**
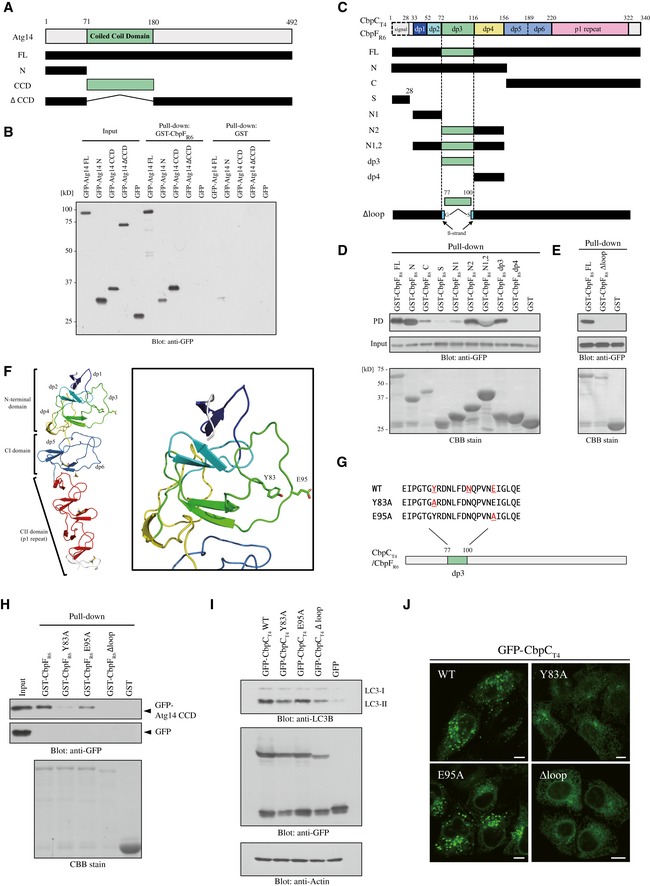
The loop structure in the CbpC dp3 domain enabled interaction with the CCD of Atg14 ADiagram of the Atg14 derivatives used in (B).BGST‐pulldown assays performed using GST‐CbpF_R6_ or GST and lysates from 293T cells expressing GFP‐Atg14 derivatives or GFP. The bound proteins were analyzed by immunoblotting using an anti‐GFP antibody.CSchematic representation of the CbpF_R6_ derivatives used in (D) and (E). The domains are color‐coded according to the crystal structure report for CbpF_R6_ (lower left) 10.D, EGST‐pulldown assays using GST‐CbpF_R6_ derivatives and lysates from 293T cells expressing GFP‐Atg14 CCD or GFP. The bound proteins were analyzed by immunoblotting using an anti‐GFP antibody. Each GST‐CbpF_R6_ derivative‐bound bead was confirmed by Coomassie brilliant blue (CBB) staining.FStructure of CbpF_R6_ (PDB ID: 2V04). The overall structure (left) and dp3 domain (right) of homology‐modeled structures are shown. The side chains of Y83 and E95 and bound choline molecules are shown as green and yellow sticks, respectively.GThe amino acid sequences of the CbpC_T4_/CbpF_R6_ mutants used in (H), (I), and (J).HGST‐pulldown assays performed using GST‐CbpF_R6_ mutants or GST and lysates from 293T cells expressing GFP‐Atg14 CCD or GFP. The bound proteins were analyzed by immunoblotting using an anti‐GFP antibody. Each GST‐CbpF_R6_ mutant‐bound bead was confirmed by CBB staining.ILysates of 293T cells transiently expressing GFP‐CbpC_T4_ mutants or GFP were subjected to SDS–PAGE and analyzed by immunoblotting using antibodies against LC3, GFP, or actin.JConfocal microscopy images of HeLa cells transiently expressing the indicated GFP‐CbpC_T4_ mutants. Scale bars, 10 μm. Source data are available online for this figure.

**Figure EV4:**
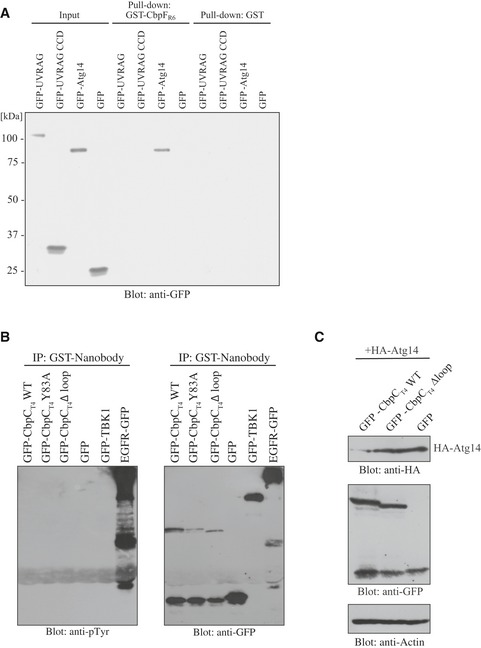
The loop structure in CbpC dp3 domain interacts with the CCD of Atg14 AGST‐pulldown assays using GST‐CbpF_R6_ or GST and lysates from 293T expressing the indicated GFP‐fused proteins or GFP were performed. Bound proteins were analyzed by immunoblotting using an anti‐GFP antibody.BLysates from 293T cells transiently expressing the indicated GFP‐fused proteins or GFP in the presence of phosphatase inhibitor were immunoprecipitated with GST‐GFP‐Nanobody and bound proteins analyzed by immunoblotting using antibodies against phosphotyrosine or GFP.CLysates from 293T cells transiently expressing HA‐Atg14, and GFP‐CbpC, CbpC Δloop, and GFP were subjected to SDS–PAGE and analyzed by immunoblotting with antibodies against HA, GFP, or actin. Source data are available online for this figure.

To identify the region of Atg14 responsible for CbpC binding, we prepared a series of GFP–Atg14 truncation mutants, including the N (residues 1–70), CCD, and ∆CCD variants, and the full‐length (FL) control Atg14 protein (Fig 4A). We then determined their abilities to interact with GST‐CbpF_R6_ by performing GST‐pulldown assays. The Atg14 CCD, which Atg14 heterodimerizes with the Beclin1 CCD 30, bound strongly to GST‐CbpF_R6_ (Fig 4B). In contrast, UVRAG CCD, which also heterodimerizes with Beclin1 through its CCD, did not bind to GST‐CbpF_R6_ (Fig EV4A). We then analyzed truncation variants of CbpF_R6_ to identify regions responsible for binding to the Atg14 CCD. Based on the structure of CbpF_R6_ 10, we designed a series of GST‐CbpF_R6_ truncation mutants, i.e., N, C, N1, N2, N1‐2, dp3, dp4, and an in‐frame deletion mutant devoid of the entire dp3 region (∆loop) (Fig 4C). We then determined their binding activities to GFP‐Atg14 CCD in GST‐pulldown assays. We found that the loop structure in the dp3 domain in the N‐terminal region of CbpF_R6_ was pivotal for Atg14 CCD binding (Fig 4D and E).

Based on the structure of CbpF_R6_ 10, we focused on two protruding loops in the dp3 domain (Fig 4F). We prepared two CbpF_R6_ mutants with single‐residue substitutions (Y83A and E95A; Fig 4F and G). Subsequent GST‐pulldown assays using these variants and lysates from 293T cells expressing GFP‐Atg14 CCD revealed that Y83 in the loop was essential for Atg14 CCD binding since the Y83A mutant (but not the E95A mutant) failed to interact with Atg14 (Fig 4H). Consistently, the accumulation of LC3‐II and intrinsic puncta formation decreased robustly in GFP‐CbpC_T4_ Y83A‐expressing cells, when compared with cells expressing E95A or WT GFP‐CbpC_T4_ (Fig 4I and J). These results suggest that the CbpC dp3–Atg14 CCD interaction promotes autophagy induction. We next addressed the possibility of CbpC Y83 phosphorylation by performing an IP experiment using GFP‐CbpC_T4_ FL, Y83A, and ∆loop in the presence of phosphatase inhibitor; however, no phosphorylation signal was detected (Fig EV4B). Furthermore, we investigated whether the CbpC dp3–Atg14 interaction functions in Atg14 degradation by transiently expressing GFP‐CbpC_T4_ ∆loop in 293T cells, and we found that Atg14 degradation was not affected by the GFP‐CbpC_T4_ ∆loop construct, which is deficient in Atg14 binding (Fig EV4C). These results also strengthen our notion that selective autophagy mediated by the Atg14–CbpC–p62 axis was involved in Atg14 degradation.

### Domain analysis of CbpC and the involvement of p62 in their interaction

**Figure 5:**
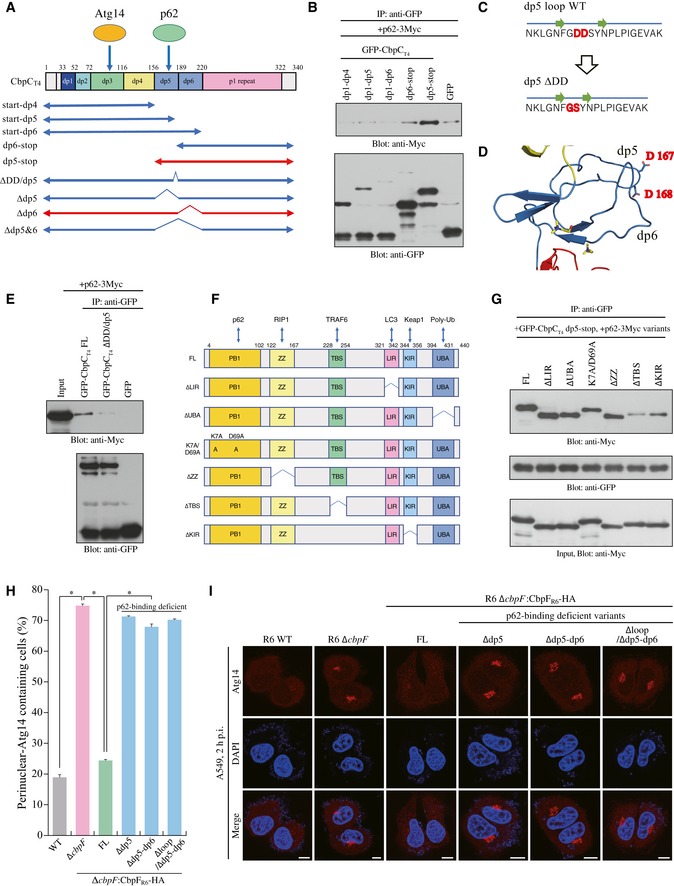
Domain analysis of the CbpC–p62 interaction ASchematic representation of the CbpC_T4_ variants used in (B), (E), (H), and (I).BLysates from 293T cells transiently expressing p62‐3Myc and GFP‐CbpC_T4_ variants were immunoprecipitated using a GST‐GFP‐Nanobody fusion protein. Bound proteins were analyzed by immunoblotting.CDiagram of the ∆DD mutation in dp5 loop domain.DStructures of the dp5 and dp6 domains in CbpF_R6_. The side chains of D167–D168 are shown.ELysates from 293T cells transiently expressing p62‐3Myc and GFP‐CbpC_T4_ variants were immunoprecipitated using the GST‐GFP‐Nanobody protein. Bound proteins were analyzed by immunoblotting.FDiagram of the p62 variants used in (G).GLysates of 293T cells transiently expressing GFP‐CbpC_T4_ and p62‐3Myc variants were immunoprecipitated with the GST‐GFP‐Nanobody protein. Bound proteins were analyzed by immunoblotting using the indicated antibodies.HA549 cells infected with the indicated *S. pneumoniae* strains for 2 h were fixed and stained with DAPI and an anti‐Atg14 antibody, and the percentages of perinuclear‐localizing Atg14 containing cells were quantified.IRepresentative epifluorescence images in (H) are shown. Scale bars, 10 μm.Data information: In (H), data represent mean ± SEM of 3 biological replicates. Student's *t*‐test was used to calculate statistical significance. *P < 0.01.Source data are available online for this figure.

**Figure EV5:**
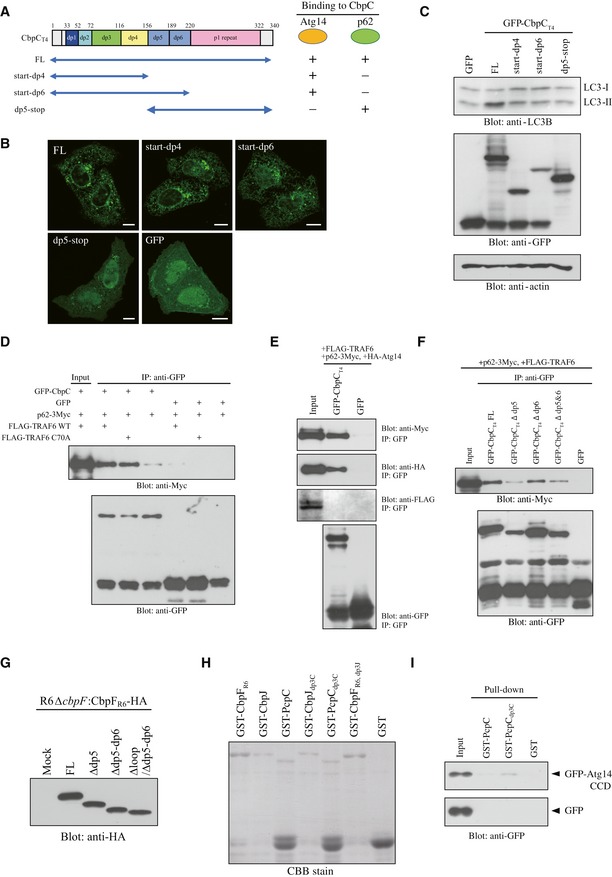
Domain analysis of CbpC–p62 interaction and comparison of Atg14‐binding capacity in CbpC family proteins ADiagram of CbpC_T4_ derivatives used in (B) and (C).BConfocal images of Hela cells transiently expressing the indicated CbpC_T4_ derivatives. Scale bars, 10 μm.CLysates from 293T cells transiently expressing the indicated CbpC_T4_ derivatives were subjected to SDS–PAGE and then analyzed by immunoblotting using antibodies against LC3B or actin.DLysates from 293T cells transiently expressing p62‐3Myc and GFP‐CbpC_T4_ in the presence or absence of FLAG‐TRAF6 (WT or E3 ligase dead) were immunoprecipitated using GST‐GFP‐Nanobody. Bound proteins were analyzed by immunoblotting.ELysates from 293T cells transiently expressing GFP‐CbpC_T4_ or GFP and p62‐3Myc and FLAG‐TRAF6 were immunoprecipitated using GST‐GFP‐Nanobody. Additionally, beads were mixed with lysates from 293T cells transiently expressing HA‐Atg14, and bound proteins were analyzed by immunoblotting.FLysates from 293T cells transiently expressing p62‐3Myc, GFP‐CbpC_T4_ variants, and FLAG‐TRAF6 were immunoprecipitated using GST‐GFP‐Nanobody. Bound proteins were analyzed by immunoblotting.GLysates from R6 ∆*cbpF* expressing the indicated CbpF_R6_‐HA variants were subjected to SDS–PAGE and analyzed by immunoblotting using an anti‐HA antibody.HEach indicated GST‐fused protein‐bound bead used in Fig 6D, H, I was confirmed by CBB staining.IGST‐pulldown assays using the indicated GST‐fused proteins and lysates from 293T expressing GFP‐Atg14 CCD or GFP. Bound proteins were analyzed by immunoblotting using an anti‐GFP antibody. Source data are available online for this figure.

We performed a domain analysis of CbpC in terms of p62 binding by preparing truncated versions of CbpC_T4_ (Fig 5A). We found that the dp5 loop of CbpC was responsible for p62 binding (Fig 5A and B). Subsequent IP assays revealed that residues D167–D168 in the dp5 loop were required for the CbpC–p62 interaction (Fig 5C–E). Therefore, we examined whether the CbpC–p62 interaction was involved in autophagy activation by employing truncated versions of CbpC, which were deficient in p62‐binding (Fig EV5A). Our data revealed that the CbpC–p62 interaction was required for intrinsic puncta formation by CbpC and autophagy activation (Fig EV5B and C). Intriguingly, intracellular localization of CbpC in the vicinity of the endoplasmic reticulum was observed only with Atg14‐interacting CbpC variants (Fig EV5B). Together, these results also support our notion that Atg14–CbpC–p62 complex formation is required for autophagy activation.

We next performed a domain analysis of p62 in terms of CbpC binding (Fig 5F). We found that the CbpC‐binding capacity decreased dramatically with the TRAF6‐binding‐deficient mutant, but only decreased partially with the Keap1‐binding‐deficient mutant (Fig 5G). These results suggested the possible involvement of TRAF6 in the CbpC–p62 interaction. We therefore examined whether the CbpC–p62 interaction was facilitated by the presence of TRAF6. IP assays using 293T cells co‐expressing GFP‐CbpC_T4_ and p62‐3Myc in the presence or absence of FLAG‐TRAF6 revealed that the CbpC–p62 interaction was robustly facilitated by the presence of TRAF6, regardless of its E3 ligase activity (Fig EV5D). However, no direct interaction of TRAF6 with the Atg14–CbpC–p62 complex was detected (Fig EV5E). Furthermore, the CbpC–p62 interaction robustly decreased with CbpC_T4_ ∆dp5, even in the presence of TRAF6 (Fig EV5F). To investigate the physiological role of the CbpC–p62 interaction via CbpC dp5, we constructed *S. pneumoniae* R6 ∆*cbpF* derivatives complemented with a series of p62‐interaction‐deficient CbpF_R6_ variants via homologous recombination, and we conducted Atg14‐degradation assays using A549 cells. Notably, we found that the disappearance of perinuclear Atg14 caused by *S. pneumoniae* infection was dramatically attenuated in *S. pneumoniae* strains expressing p62‐binding‐deficient CbpF_R6_ (Figs 5H and I, and EV5G).

### Autophagy induction activity via Atg14 binding was a distinctive property of CbpC_T4_ and CbpF_R6_ from their orthologs

**Figure 6:**
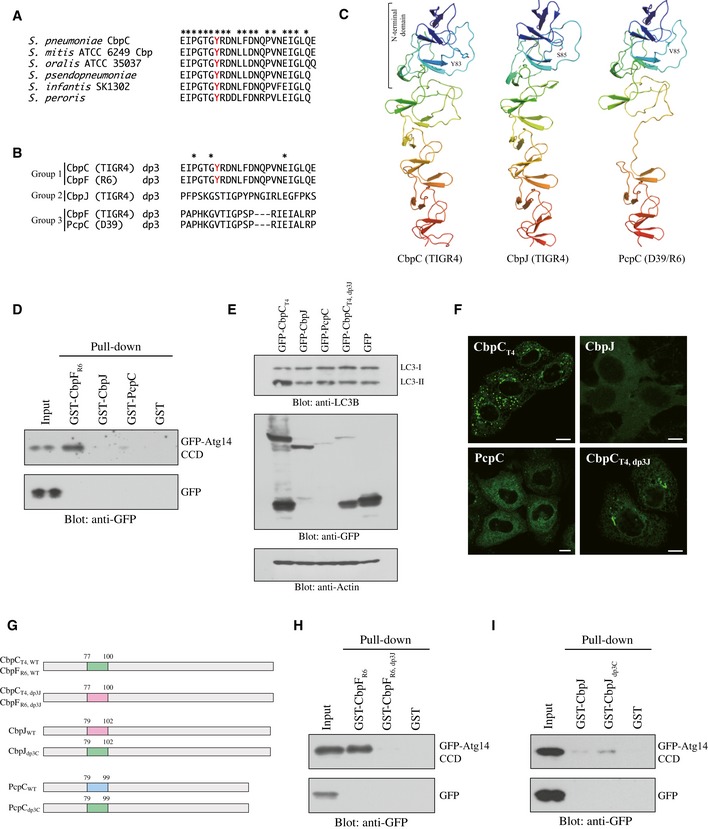
Comparison of the Atg14‐binding capacities and autophagy induction activities of CbpC family proteins AComparison of the amino acid sequences of the dp3 domains from CbpC_T4_ orthologs of other *Streptococcus* strains. The identical amino acid residues in all sequences in (A) and (B) are indicated by asterisk.BComparison of the amino acid sequences of the dp3 domains in CbpC_T4_ orthologs CbpJ, CbpF_T4_, CbpF_R6_, and PcpC.CFull‐length structures of CbpC_T4_ (PDB ID: 2V04), CbpJ (PDB ID: 6JYX), and PcpC (homology model).DGST‐pulldown assays using the indicated GST‐fusion proteins or GST and lysates from 293T cells expressing GFP‐Atg14 CCD or GFP. Bound proteins were analyzed by immunoblotting using an anti‐GFP antibody.ELysates from 293T cells expressing GFP‐fusion proteins or GFP were subjected to SDS–PAGE and analyzed by immunoblotting using antibodies against LC3, GFP, or actin.FConfocal images of HeLa cells transiently expressing GFP‐fusion proteins. Scale bars, 10 μm.GDiagram of CbpC_T4_/CbpF_R6_, CbpJ, and PcpC dp3 domain swaps with CbpJ or CbpC_T4_/CbpF_R6_.H, IGST‐pulldown assays using the indicated GST‐fused proteins or GST and lysates from 293T expressing GFP‐Atg14 CCD or GFP. Bound proteins were analyzed by immunoblotting using an anti‐GFP antibody. Source data are available online for this figure.

Basic Local Alignment Search Tool (BLAST) analysis suggested that CbpC_T4_ orthologs exist in multiple *Streptococcus* strains, including *S. mitis* and *S. oralis* (Fig 6A). Furthermore, the *S. pneumoniae* TIGR4 strain has two CbpC_T4_ orthologs (CbpJ and CbpF_T4_), and the R6 strain also has two CbpC_T4_‐orthologs (CbpF_R6_ and PcpC), as shown in Figs 6B and EV2E 10. As the amino acid sequences of dp3 in these homologs were distinct, we classified them into three groups (Fig 6B). The crystal structure of CbpJ 11 and a homology model of PcpC were compared with the structure of CbpF_R6_ (Fig 6C). While PcpC lacks the N‐terminal portion of the CBR (Fig EV2E), homology modeling indicated that both CbpJ in TIGR4 and PcpC in R6 have retained the basic structure of the dp3 domain. However, the loops of CbpJ and PcpC, which contain residues corresponding to Y83 (S85 in CbpJ and V85 in PcpC), have distinct structures from that of CbpF_R6_.

We then examined the binding capacity of CbpJ from TIGR4 (Group 2, Fig 6B) and PcpC from R6 (Group 3, Fig 6B) to Atg14 CCD by performing GST‐pulldown assays. We found that CbpJ and PcpC were unable to bind Atg14 (Figs 6D and EV5H). The increase in LC3‐II protein level and puncta formation was not observed in GFP‐CbpJ‐ or GFP‐PcpC‐expressing cells (Fig 6E and F), showing that autophagy induction by Atg14 CCD binding was an intrinsic property of CbpC_T4_ (Group 1).

We next prepared dp3 domain‐swapped proteins (Figs 6G and EV5H) and measured their binding to the Atg14 CCD in GST‐pulldown assays. Interestingly, the Atg14‐binding capacity was abolished with CbpF_R6, dp3J_, where dp3 from CbpF_R6_ was swapped with dp3 from CbpJ (Fig 6H). The increase in LC3‐II protein level and intrinsic puncta formation was also abolished in cells expressing CbpC_dp3J_, where dp3 from CbpC_T4_ was swapped with dp3 from CbpJ (Fig 6E and F). These results also suggest that the CbpC–Atg14 CCD interaction was required for autophagy induction. The Atg14 CCD‐binding abilities of CbpJ_dp3C_ (dp3 in CbpJ swapped with dp3 from CbpC_T4_) and PcpC_dp3C_ (dp3 in PcpC was swapped with dp3 from CbpC_T4_) were partially restored (Figs 6I and EV5I). Together, these results strongly suggest that autophagy induction through Atg14 binding was an intrinsic property of the Group 1‐CbpC_T4_ family.

## Discussion

In this study, we identified CbpC from the *S. pneumoniae* TIGR4 strain as an autophagy‐activating virulence factor. We found that CbpC binding to Atg14 was required for autophagy activation. Our data further revealed that selective autophagy triggered by the Atg14–CbpC–p62 axis promoted the autophagic degradation of Atg14, resulting in suppressed autophagosome–lysosome fusion, which contributes to the intracellular viability of *S. pneumoniae*.

To understand these phenomena in more detail, we demonstrated that dp3 in the N‐terminal region of CbpC was responsible for its binding to the CCD of Atg14. Further domain analysis of Atg14 binding to CbpC revealed that the Y83 residue in dp3 was crucial for Atg14 CCD binding. Indeed, the Y83A and dp3 loop‐deleted CbpC mutants were deficient in autophagy induction. The N‐terminal region is also involved in binding to LytC, a muramidase involved in pneumococcal autolysis 10. LytC acts as a lysozyme during growth at 30°C, and the CbpF_R6_–LytC interaction suppresses autolysis 10. It is not yet known whether the binding of LytC and CbpC_T4_ to the N‐terminal region of Atg14 is competitive. Our findings also revealed that CbpC can interact with p62 via the dp5 domain. The CbpC–p62 interaction was dramatically promoted by TRAF6. CbpC showed no binding capacity for TRAF6. The molecular mechanism whereby TRAF6 promotes the CbpC–p62 interaction remains to be elucidated.

The *S. pneumoniae* TIGR4 strain has two CbpC_T4_ orthologs, namely CbpJ and CbpF_T4_; the R6 strain also has two CbpC_T4_‐orthologs, namely CbpF_R6_ and PcpC (Figs 6B and EV2E) 10. The coexistence of these two highly similar orthologs in the TIGR4 and R6 strains indicates the functional correlation of these genes. These pneumococcal proteins have the same architecture as CbpC_T4_ and are likely to constitute a CbpC_T4_‐like subfamily. They each have a typical CBM in their C‐terminal domain and a similar structural framework in their N‐terminal domain (composed of several non‐consensus CBRs), as well as distinctive insertion sequences. These intrinsic insertions between two β‐strands form connecting loops and might provide each CbpC_T4_‐like protein with an intrinsic physiological function. Here, we showed that dp3 and dp5 from CbpC can bind to Atg14 and p62, respectively; however, the Atg14‐binding capacity was not seen in dp3 from CbpJ and PcpC. Although the binding partner of dp3 and dp5 from CbpJ and PcpC remains unknown, it is can be speculated that the CbpC_T4_‐like subfamily might serve a similar intracellular function as that of CbpC_T4_.

In mammalian cells, Atg14 levels are controlled by ZBTB16 E3 ligase via G‐protein‐coupled receptor (GPCR) stimulation 31. Upon GPCR stimulation, ZBTB16 promotes Atg14 degradation to inhibit autophagy. Here, we demonstrated a sophisticated pathogen‐driven Atg14‐degradation mechanism. Intriguingly, *S. pneumoniae* utilized host‐selective autophagy to reduce Atg14, resulting in the suppression of continuous autophagic activation and autophagosome–lysosome fusion. Atg14 has two functions in autophagy. During the initiation of autophagy, Atg14 acts as an autophagy‐specific regulator of the class III phosphatidylinositol 3‐kinase complex (PI3KC3) to generate PI3P 24. During autophagy maturation, Atg14 promotes STX17–SNAP29–VAMP8‐mediated autophagosome fusion with lysosomes 27. In this study, we demonstrated that CbpC has two distinct effects on autophagy: It activates autophagy initiation and suppresses autophagosome–lysosome fusion. Ectopic CbpC expression increased autophagy flux and elevated LC3‐II levels, even in the presence of chloroquine. However, it also caused subsequent Atg14 degradation and attenuated autophagic degradation. This duality is reminiscent of the effects of the VacA toxin of *Helicobacter pylori* on autophagic activation 32. A short period of VacA treatment (6 h) triggers autophagy; however, more prolonged VacA treatment (24 h), similar to the conditions observed during chronic infection, can inhibit autophagic degradation by disrupting autophagic flux. We demonstrated that CbpC inhibited autophagic flux; however, a detailed time course of the changes of CbpC‐driven autophagic activation and subsequent Atg14 depletion during pneumococcal infection has not been fully revealed. Furthermore, prolonged Atg14 depletion can prevent not only on autophagosome–lysosome fusion, but also continuous autophagic induction. In this study, we did not clarify whether CbpC_T4_‐driven Atg14 depletion suppressed both autophagy activation and autophagosome–lysosome fusion. It is captivating to speculate that intracellular *S. pneumoniae* can establish prolonged or chronic infection by deploying CbpC to subvert both autophagic induction and degradation.

PI3K3C3 forms two mutually exclusive complexes that execute distinct functions 25, 26. One complex is with Atg14 and promotes autophagy initiation, whereas the other complex replaces Atg14 with UVRAG–Rubicon and suppresses autophagy. Considering that CbpC cannot bind to the CCD domain of UVRAG (Fig EV4A), we conclude that CbpC‐triggered autophagy is unlikely due to inhibited UVRAG–Beclin1 complex formation or promotion of the Atg14–Beclin1 interaction.

Many pathogens suppress autophagosome–lysosome fusion by targeting SNARE proteins, such as SNAP29 and Stx17. The 3C protease from enterovirus D68 cleaves the autophagic SNARE protein, SNAP29, to inhibit autophagic flux and to promote viral replication 33. The 3C protease from coxsackievirus B3 cleaves SNAP29 and PLEKHM1 to facilitate its own propagation 34. Human parainfluenza virus, type 3 deploys a phosphoprotein to inhibit autophagic flux 35. The phosphoprotein interferes with the SNAP29–Stx17 interaction, resulting in the prevention of autophagosome–lysosome fusion. Lpg1137 from *Legionella pneumophila*, an effector protein acting as a serine protease, can cleave Stx17 during *Legionella* infection to inhibit autophagic flux 17. In this study, we revealed novel bacterial tactics for targeting Atg14 to dampen autophagic degradation. *S. pneumoniae* hijacks host autophagy to deplete Atg14 by deploying CbpC.

In summary, we showed that *S. pneumoniae* manipulates the selective autophagy system to spatiotemporally regulate the level of Atg14 and dampening antibacterial xenophagy induced at later stages of infection, thereby increasing its viability and ability to invade more deeply into host tissues. Our discovery provides insight into the battles between intracellular pulmonary bacterial pathogens and host‐defense systems; it may facilitate the development of therapeutic targets for controlling pneumococcal diseases.

## Materials and Methods

### Bacterial strains

The *S. pneumoniae* strains R6 (ATCC BAA‐255) and TIGR4 (ATCC BAA‐334) were purchased from the American Type Culture Collection. *S. pneumoniae* were grown in standing cultures of Todd–Hewitt Broth (THY; Becton Dickinson [BD], San Jose, CA, USA) containing 0.5% yeast extract (BD) broth or were plated on THY agar plates or Columbia agar plates with 5% sheep blood (BD) at 37°C in 5% CO_2_. The *E. coli* strains MC1061, DH10B, BL21, and C43 (Cosmo Bio, Tokyo, Japan) were used for DNA cloning and protein expression, and were grown in LB medium or on LB‐agar plates supplemented with 100 μg/ml ampicillin or 50 μg/ml kanamycin.

### Reagents and antibodies

Antibodies against GFP (D5.1), phosphotyrosine (P‐Tyr‐100), and LC3A/B (D3U4C) (Cell Signaling Technology, Massachusetts, USA); Myc (A14) and β‐actin antibodies (C4, Santa Cruz Biotechnology, Texas, USA); GM130 (35/GM130, BD); HA (TANA2), Atg14 (PD026), Atg16L1 (M150‐3), p62 (PM045), and poly‐Ub (FK2) (MBL, Nagoya, Japan); and FLAG (FUJIFILM Wako Pure Chemical, Osaka, Japan) were used as primary antibodies. Horseradish peroxidase‐conjugated goat anti‐rabbit or anti‐mouse antibodies (The Jackson Laboratory, Maine, USA) were used as secondary antibodies in immunoblotting experiments. Anti‐Myc (9B11, Cell Signaling Technology), Protein G PLUS‐Agarose (sc‐2002, Santa Cruz Biotechnology), EZview Red Anti‐HA Affinity Gel (Sigma, MO, USA), and GFP‐Trap (Chromotech, Planegg‐Martinsried, Germany) were used for IP experiments. Autophagy was induced and inhibited by 10 μM rapamycin, 40 μM chloroquine (Selleck Chemical, Texas, USA), and 100 nM Bafilomycin A1 (AdipoGen, CA, USA). Cycloheximide (50 μg/ml, FUJIFILM Wako Pure Chemical) was used to inhibit protein synthesis. All other reagents were purchased from FUJIFILM Wako Pure Chemical.

### Plasmids

Polymerase chain reaction (PCR) experiments were performed with Q5 High‐Fidelity DNA Polymerase (New England BioLabs, Massachusetts, USA), and cDNA for reverse transcriptase‐PCR (RT–PCR) experiments was synthesized using the SuperScript III One‐Step RT‐PCR System with Platinum Taq (Thermo Fisher Scientific, Massachusetts, USA). To generate the pEGFP‐Cbp constructs, the *cbp* and *lytR* genes of *S. pneumoniae* TIGR4 and R6, and the *pspC* gene of *S. pneumoniae* R6 were subcloned into pEGFP‐C1 (Takara Bio, Shiga, Japan) and pGEX6P‐1 (GE Healthcare, Illinois, USA). Rat LC3B cDNA was subcloned into pmCherry‐C1 (Takara Bio). Human Atg14 and TBK1 cDNAs were subcloned into pEGFP‐C1. Human EGFR cDNA was subcloned into pEGFP‐N3. Myc‐tagged human Atg14 cDNA was subcloned into pcDNA3.1 (Thermo Fisher Scientific) for expression in mammalian cells or pTB101 36 for expression in *E. coli* cells. Hemagglutinin (HA)‐tagged human Atg14 cDNA was subcloned into pMXs‐puro (Cosmo Bio) or pcDNA6.2 (Thermo Fisher Scientific). GFP and GFP‐CbpC cDNAs were subcloned into pMXs‐puro or pMXs‐blast. Mammalian expression vectors for pIgR, Atg5‐3Myc, p62‐3Myc, GFP‐Atg5, GFP‐Atg12, GFP‐Atg16L1, GFP‐Beclin1, GFP‐WIPI1, and GFP‐WIPI2 were constructed previously 18, 37. Human Atg14 cDNA was subcloned into pHTN HaloTag and pNLF1‐N (Promega, Wisconsin, USA), human Stx17 cDNA was subcloned into pHTN HaloTag, and human Beclin1 cDNA was subcloned into pNLF1‐N. Plasmids encoding Atg14 ∆CCD, CbpC (∆loop, Y83A, E95A), and swapped CbpC, CbpJ, and PcpC derivatives were designed using NEBuilder (New England BioLabs). The GST‐GFP‐Nanobody expression vector 38 was a generous gift from Dr. Yohei Katoh (Kyoto University). The sequences of the primers used in this study are shown in Appendix Table S1.

### Cell culture and transfection

The HeLa (human cervical cancer), 293T and 293A (human embryonic kidney fibroblasts), A549 (human lung epithelial cells), and MEF (mouse embryonic fibroblasts) cell lines 39, 40 were cultured in Dulbecco's modified Eagle's medium (DMEM, Nakalai Tesque, Kyoto, Japan) supplemented with 10% fetal calf serum (FCS; Gibco‐Thermo Fisher Scientific, Massachusetts, USA), 100 μg/ml gentamicin (FUJIFILM Wako Pure Chemical), and 60 μg/ml kanamycin (FUJIFILM Wako Pure Chemical). For retroviral production, platE cells were maintained in DMEM with 10% FCS, 1 μg/ml puromycin (Sigma), and 10 μg/ml blasticidin (Kaken Pharmaceutical, Tokyo, Japan). MEF‐derived stable clones were cultured in DMEM containing 10% FCS, 1 μg/ml puromycin, and/or 10 μg/ml blasticidin. Transfections were performed with PEI MAX (Polysciences, Pennsylvania, USA), Lipofectamine LTX (Thermo Fisher Scientific), or the Effectene Transfection Reagent (Qiagen, Rhine‐Westphalia, Germany), according to the manufacturers’ protocols.

### Fluorescence microscopy

HeLa cells expressing GFP‐Cbps were fixed with 4% paraformaldehyde in phosphate buffer (FUJIFILM Wako Pure Chemical) for 15 min at room temperature and washed three times with phosphate‐buffered saline (PBS). After washing with distilled water, the specimens were mounted with Vector Shield (Vector Laboratories, CA, USA) and analyzed by confocal microscopy (LSM700, Zeiss, Baden‐Württemberg, Germany).

### Recombinant retroviruses and infections

Recombinant retroviruses were prepared as previously described 19, 20. Briefly, retroviral plasmids were transfected into platE cells for 2 days, after which time the culture supernatant containing retrovirus was collected and centrifugated. Cleared supernatant was used for the retroviral infections. For retroviral infection, recipient cells were infected with retroviruses by adding cleared supernatant in the presence of polybrene for 6 h. Stable transformants were selected in DMEM/10% FCS with 1 μg/ml puromycin or 10 μg/ml blasticidin.

### Small interfering RNA (siRNA) experiments

siRNAs were synthesized and duplexed by siRNA Co., Ltd (Tokyo, Japan). The sequences of siRNAs targeting human pIgR, Atg5, or p62 mRNA are shown in Appendix Table S2. The siRNAs were reverse transfected into cells using Lipofectamine RNAi MAX (Thermo Fisher Scientific), according to the manufacturer's protocol. The knockdown efficiency was checked by RT–PCR using the primer pairs shown in Appendix Table S2.

### Construction of the *S. pneumoniae* mutants and complemented strains

Target gene inactivation in *S. pneumoniae* strain TIGR4 or R6 was performed as described previously 20. Briefly, an *erm* cassette with long flanking regions homologous to the target gene was generated using two‐step PCR as described 41, using the primers listed in Appendix Table S3. The PCR products were introduced into competent *S. pneumoniae* TIGR4 or R6 cells as described previously 20, and the transformants were selected in 1 μg/ml erythromycin. Substitution of the target gene was confirmed by PCR with primers shown in Appendix Table S3. The *S. pneumoniae* ∆*ply* strain was previously reported 20. To construct the *S. pneumoniae* R6 ∆*cbpF*/pCbpF‐FLAG strain, a PCR product encoding the c*bpF*_*R6*_ *‐FLAG* gene and its promoter was generated by PCR using the primers shown in Appendix Table S4. The PCR product was inserted into pMX1 42, and the ligated plasmid was introduced into competent *S. pneumoniae* R6 ∆*cbpF* cells, as described previously 20, and the transformants were selected in 50 μg/ml spectinomycin. Target gene expression was confirmed by Western blotting. *S. pneumoniae* R6 ∆*cbpF* derivatives were complemented with a series of p62‐interaction‐deficient CbpF_R6_ variants by homologous recombination. A *cat* cassette with *cbpF*_*R6*_ *‐HA* variants and long flanking regions homologous to the target locus was generated using two‐step PCR as described 41 with the primers shown in Appendix Table S4. The PCR products were introduced into competent cells of the *S. pneumoniae* strain R6 as described previously 20, and transformants were selected in 2.5 μg/ml chloramphenicol. Substitution of the target gene was confirmed by PCR with the primers shown in Appendix Table S4. The primers were designed to target *cbpF*_*R6*_ *‐HA* variants into the *cps2H* gene (which is not expressed in the R6 strain) in reverse orientation relative to the *cps* operon.

### *In vitro* protein production and binding assays

Wheat germ cell‐free protein production was performed as described previously 43. Briefly, cDNA templates encoding FLAG or biotin ligase site (bls) epitopes were generated by PCR with split‐primer sets for autophagy‐related genes. Amplified cDNAs were then used to produce proteins by the bilayer method using the WEPRO1240 Expression Kit (CellFree Sciences, Ehime, Japan). Biotinylated CbpC or control GFP proteins were mixed with FLAG‐tagged autophagy‐related proteins and incubated for 2 h at 26°C in different wells of a 96‐well plate. Next, 20 μl of pre‐buffered Streptavidin MagneSphere Paramagnetic Particles (Promega) was added to each well and incubated for 30 min at room temperature, and then washed three times with 120 μl of PBS/0.1% NP‐40. The beads were then suspended in 20 μl of 2× SDS sample buffer and boiled for 5 min for sodium dodecyl sulfate–polyacrylamide gel electrophoresis (SDS–PAGE) and immunoblotting analysis.

### GST‐GFP‐Nanobody protein purification

*Escherichia coli* BL21 cells harboring the pGST‐GFP‐Nanobody plasmid were cultured in LB medium with 50 μg/ml ampicillin for 2 h at 37°C. Isopropyl‐1‐thio‐β‐D‐galactopyranoside (IPTG) was added to the culture medium at a final concentration of 0.1 mM. After incubation for 2 h at 37°C, the bacteria were harvested. To express GST‐Cbps, *E. coli* cells were cultured at 20°C for 9 h, after which 0.1 mM IPTG was added and the bacteria were incubated at 20°C for an additional 18 h. Following IPTG induction, the bacteria were harvested by centrifugation (4,300 *g*, 10 min, 4°C) and suspended in wash buffer (Tris‐buffered saline [TBS] + 1% Triton X‐100 + 10 mM β‐mercaptoethanol) containing ethylenediaminetetraacetic acid (EDTA)‐free Complete protease‐inhibitor cocktail (Sigma). After sonication (3 × 30 s on ice) and centrifugation (4,300 *g*, 10 min, 4°C), the cleared lysates were mixed with pre‐buffered glutathione Sepharose 4B (GE Healthcare), and GST‐fusion proteins were purified according to the manufacturer's protocol.

### GST‐pulldown assays using cell lysates

293T cells were transfected with the indicated plasmids in 6‐well plates, suspended in 500 μl of wash buffer (TBS/0.5% NP‐40) containing EDTA‐free Complete protease‐inhibitor cocktail, sonicated on ice for 10 s, and centrifuged at 16,900 *g* at 4°C for 10 min. Five microliters of GST‐GFP‐Nanobody bound to glutathione Sepharose 4B was mixed with cleared 293T cell lysates and incubated with rotation for 2 h at 4°C. The beads were washed three times with 1 ml of wash buffer and suspended in 30 μl of 2× SDS sample buffer and boiled for 7 min. The bound proteins were analyzed by immunoblotting.

### IP from cell lysates

Cells (293T) were seeded into 6‐well plates and transfected with the indicated plasmids. After 18 h, the cells were suspended in 500 μl of wash buffer (TBS with 5 mM MgCl_2_, 1 mM EDTA, and 0.05% NP‐40) containing Complete protease‐inhibitor cocktail, sonicated on ice for 10 s, and centrifuged at 16,900 × *g* at 4°C for 10 min. Five microliters of GST‐GFP‐Nanobody‐bound glutathione Sepharose 4B beads, GFP‐Trap, or EZview Red Anti‐HA Affinity Gel were mixed with the cleared lysates and rotated for 2 h at 4°C. In phosphorylation assays, IP was performed using GFP‐Trap in the presence of a phosphatase inhibitor (PhosphoStop, Sigma). IPs with an anti‐Myc antibody with Protein G PLUS‐Agarose were performed according to the manufacturers’ protocols using IP buffer (50 mM Tris‐HCl pH 7.4, 150 mM NaCl, 1 mM EDTA, 10% glycerol, and Complete protease‐inhibitor cocktail‐EDTA). The beads were washed three times with 1 ml of wash buffer, suspended in 30 μl of 2× SDS sample buffer, and boiled for 7 min. The bound proteins were analyzed by immunoblotting.

### Infecting cells with *Streptococcus pneumoniae* for Western blotting

MEFs/HA‐Atg14/pIgR were infected with *S. pneumoniae* as previously described 20, 44, 45. Briefly, MEFs/HA‐Atg14 cells (seeded at 2 × 10^5^ cells/well in 6‐well plates) were infected with fresh *S. pneumoniae* at a multiplicity of infection of 100 and then centrifuged at 1,000 rpm for 5 min at room temperature. The cells were then incubated for 1 h at 37°C in 5% CO_2_ and washed three times with Hanks’ balanced salt solution (HBSS). DMEM containing 10% FCS, 200 μg/ml gentamicin, and 200 U/ml catalase was added to each well, and the cells were incubated for 30 min to kill extracellular bacteria. After changing the medium to DMEM with 10% FCS, 100 μg/ml gentamicin, and 200 U/ml catalase (incubation buffer), the cells were incubated for the indicated periods at 37°C in 5% CO_2_. Unless otherwise stated, inhibitors were included in the incubation buffer to avoid affecting the bacterial invasion efficiency. At each time point, the cells were lysed with 100 μl of 2× SDS sample buffer, and the lysate was boiled for 7 min. After sonication (10 × 1 s), equal volumes of lysates were separated by SDS–PAGE and analyzed by Western blotting using the indicated antibodies.

### NanoBRET assays

Cells (293A) in 96‐well plates were transfected with the NanoLuc and HaloTag expression vectors at a 1:100 ratio in the presence or absence of GFP‐CbpC‐ or GFP‐expression vectors. At 48 h post‐transfection, NanoBRET activity was measured using the NanoBRET Nano‐Glo Detection System (Promega), as previously described 29.

### Intracellular bacterial‐survival assay

Intracellular bacterial‐survival assays were performed as described previously 20. Briefly, MEFs/pIgR seeded on 24‐well plates were infected with WT or ∆*cbpC S. pneumoniae*, as described above. After centrifugation, the cells were incubated for 1 h at 37°C in 5% CO_2_, washed twice with HBSS, and then incubated for 15 min with 500 μl of DMEM containing 10% FCS, 200 μg/ml gentamicin, and 200 U/ml catalase. To kill extracellular bacteria, the cells were incubated for another 15 min with DMEM containing 10% FCS, 200 μg/ml gentamicin, 200 U/ml catalase, and 10 μg/ml penicillin G (FUJIFILM Wako Pure Chemical). After washing the cells three times with HBSS, they were incubated with DMEM containing 10% FCS and 200 U/ml catalase for the indicated periods at 37°C in 5% CO_2_, after which they were lysed in PBS with 1.0% saponin (Sigma). The lysates were serially diluted with PBS containing 0.1% saponin onto THY agar plates, and the number of intracellular bacteria was expressed as the number of colony‐forming units (CFUs).

### Homology modeling

Homology modeling of CbpC from *S. pneumoniae* TIGR4 (locus tag: SP_0377) was performed using the SWISS‐MODEL server 46. The crystal structure of the mature CbpF protein from *S. pneumoniae* R6 (locus tag: SPR0337), which has 92.3% amino acid‐sequence identity with SP_0377 10, was used as a template. The GMQE and QMEAN values were 0.99 and 1.06, respectively, suggesting that the homology modeling was reliable.

### Quantification and statistical analysis

Data are shown as the mean ± standard error of the mean (SEM). *P* values were calculated using Student's *t*‐test. If the standard deviation values were significantly different (*F* < 0.05), then *P* values were calculated using the Mann–Whitney *U* test with Prism 6 software.

## Author contributions

MOg, AR, and MO supervised the overall project. MOg, SS, SM, SF, and AR designed the experiments. SS, MOg, SM, and MT performed the experiments. SS, MOg, SM, MT, TI, SF, AR, and MOg analyzed the data. MOg wrote the manuscript with input from all co‐authors.

## Conflict of interest

The authors declare that they have no conflict of interest.

## Supporting information



Appendix

Expanded View Figures PDF

Source data for Expanded View

Review Process File

Source data for Figure 1

Source data for Figure 2

Source data for Figure 3

Source data for Figure 4

Source data for Figure 5

Source data for Figure 6

## References

[1] Henriques‐Normark B , Tuomanen EI (2013) The pneumococcus: epidemiology, microbiology, and pathogenesis. Cold Spring Harb Perspect Med 3: a010215 10.1101/cshperspect.a010215 PMC3685878 23818515

[2] van der Poll T , Opal SM (2009) Pathogenesis, treatment, and prevention of pneumococcal pneumonia. Lancet 374: 1543–1556 19880020 10.1016/S0140-6736(09)61114-4

[3] Nakano S , Fujisawa T , Ito Y , Chang B , Suga S , Noguchi T , Yamamoto M , Matsumura Y , Nagao M , Takakura S *et al* (2016) Serotypes, antimicrobial susceptibility, and molecular epidemiology of invasive and non‐invasive *Streptococcus pneumoniae* isolates in paediatric patients after the introduction of 13‐valent conjugate vaccine in a nationwide surveillance study conducted in Japan in 2012–2014. Vaccine 34: 67–76 26602268 10.1016/j.vaccine.2015.11.015

[4] Hakenbeck R , Madhour A , Denapaite D , Bruckner R (2009) Versatility of choline metabolism and choline‐binding proteins in *Streptococcus pneumoniae* and commensal streptococci. FEMS Microbiol Rev 33: 572–586 19396958 10.1111/j.1574-6976.2009.00172.x

[5] Kadioglu A , Weiser JN , Paton JC , Andrew PW (2008) The role of *Streptococcus pneumoniae* virulence factors in host respiratory colonization and disease. Nat Rev Microbiol 6: 288–301 18340341 10.1038/nrmicro1871

[6] Frolet C , Beniazza M , Roux L , Gallet B , Noirclerc‐Savoye M , Vernet T , Di Guilmi AM (2010) New adhesin functions of surface‐exposed pneumococcal proteins. BMC Microbiol 10: 190 20624274 10.1186/1471-2180-10-190 PMC2911433

[7] Maestro B , Sanz JM (2016) Choline binding proteins from *Streptococcus pneumoniae*: a dual role as enzybiotics and targets for the design of new antimicrobials. Antibiotics (Basel) 5: E21 27314398 10.3390/antibiotics5020021 PMC4929436

[8] Perez‐Dorado I , Galan‐Bartual S , Hermoso JA (2012) Pneumococcal surface proteins: when the whole is greater than the sum of its parts. Mol Oral Microbiol 27: 221–245 22759309 10.1111/j.2041-1014.2012.00655.x

[9] Hermoso JA , Lagartera L , Gonzalez A , Stelter M , Garcia P , Martinez‐Ripoll M , Garcia JL , Menendez M (2005) Insights into pneumococcal pathogenesis from the crystal structure of the modular teichoic acid phosphorylcholine esterase Pce. Nat Struct Mol Biol 12: 533–538 15895092 10.1038/nsmb940

[10] Molina R , Gonzalez A , Stelter M , Perez‐Dorado I , Kahn R , Morales M , Moscoso M , Campuzano S , Campillo NE , Mobashery S *et al* (2009) Crystal structure of CbpF, a bifunctional choline‐binding protein and autolysis regulator from *Streptococcus pneumoniae* . EMBO Rep 10: 246–251 19165143 10.1038/embor.2008.245 PMC2658566

[11] Xu Q , Zhang JW , Chen Y , Li Q , Jiang YL (2019) Crystal structure of the choline‐binding protein CbpJ from *Streptococcus pneumoniae* . Biochem Biophys Res Commun 514: 1192–1197 31104766 10.1016/j.bbrc.2019.05.053

[12] Gutierrez‐Fernandez J , Saleh M , Alcorlo M , Gomez‐Mejia A , Pantoja‐Uceda D , Trevino MA , Voss F , Abdullah MR , Galan‐Bartual S , Seinen J *et al* (2016) Modular architecture and unique teichoic acid recognition features of choline‐binding protein L (CbpL) contributing to pneumococcal pathogenesis. Sci Rep 6: 38094 27917891 10.1038/srep38094 PMC5137146

[13] Li Q , Cheng W , Morlot C , Bai XH , Jiang YL , Wang W , Roper DI , Vernet T , Dong YH , Chen Y *et al* (2015) Full‐length structure of the major autolysin LytA. Acta Crystallogr D Biol Crystallogr 71: 1373–1381 26057677 10.1107/S1399004715007403

[14] Kohler LJ , Roy CR (2017) Autophagic targeting and avoidance in intracellular bacterial infections. Curr Opin Microbiol 35: 36–41 27984783 10.1016/j.mib.2016.11.004 PMC5963723

[15] Galluzzi L , Baehrecke EH , Ballabio A , Boya P , Bravo‐San Pedro JM , Cecconi F , Choi AM , Chu CT , Codogno P , Colombo MI *et al* (2017) Molecular definitions of autophagy and related processes. EMBO J 36: 1811–1836 28596378 10.15252/embj.201796697 PMC5494474

[16] Stolz A , Ernst A , Dikic I (2014) Cargo recognition and trafficking in selective autophagy. Nat Cell Biol 16: 495–501 24875736 10.1038/ncb2979

[17] Arasaki K , Mikami Y , Shames SR , Inoue H , Wakana Y , Tagaya M (2017) Legionella effector Lpg1137 shuts down ER‐mitochondria communication through cleavage of syntaxin 17. Nat Commun 8: 15406 28504273 10.1038/ncomms15406 PMC5440676

[18] Ogawa M , Yoshimori T , Suzuki T , Sagara H , Mizushima N , Sasakawa C (2005) Escape of intracellular *Shigella* from autophagy. Science 307: 727–731 15576571 10.1126/science.1106036

[19] Yoshikawa Y , Ogawa M , Hain T , Yoshida M , Fukumatsu M , Kim M , Mimuro H , Nakagawa I , Yanagawa T , Ishii T *et al* (2009) *Listeria monocytogenes* ActA‐mediated escape from autophagic recognition. Nat Cell Biol 11: 1233–1240 19749745 10.1038/ncb1967

[20] Ogawa M , Matsuda R , Takada N , Tomokiyo M , Yamamoto S , Shizukusihi S , Yamaji T , Yoshikawa Y , Yoshida M , Tanida I *et al* (2018) Molecular mechanisms of *Streptococcus pneumoniae*‐targeted autophagy via pneumolysin, Golgi‐resident Rab41, and Nedd4‐1‐mediated K63‐linked ubiquitination. Cell Microbiol 20: e12846 29582580 10.1111/cmi.12846

[21] Lemon JK , Weiser JN (2015) Degradation products of the extracellular pathogen *Streptococcus pneumoniae* access the cytosol via its pore‐forming toxin. MBio 6: e02110‐14 25604786 10.1128/mBio.02110-14 PMC4313911

[22] Morsczeck C , Prokhorova T , Sigh J , Pfeiffer M , Bille‐Nielsen M , Petersen J , Boysen A , Kofoed T , Frimodt‐Moller N , Nyborg‐Nielsen P *et al* (2008) *Streptococcus pneumoniae*: proteomics of surface proteins for vaccine development. Clin Microbiol Infect 14: 74–81 18034862 10.1111/j.1469-0691.2007.01878.x

[23] Kabeya Y , Mizushima N , Ueno T , Yamamoto A , Kirisako T , Noda T , Kominami E , Ohsumi Y , Yoshimori T (2000) LC3, a mammalian homologue of yeast Apg8p, is localized in autophagosome membranes after processing. EMBO J 19: 5720–5728 11060023 10.1093/emboj/19.21.5720 PMC305793

[24] Matsunaga K , Morita E , Saitoh T , Akira S , Ktistakis NT , Izumi T , Noda T , Yoshimori T (2010) Autophagy requires endoplasmic reticulum targeting of the PI3‐kinase complex via Atg14L. J Cell Biol 190: 511–521 20713597 10.1083/jcb.200911141 PMC2928018

[25] Matsunaga K , Saitoh T , Tabata K , Omori H , Satoh T , Kurotori N , Maejima I , Shirahama‐Noda K , Ichimura T , Isobe T *et al* (2009) Two Beclin 1‐binding proteins, Atg14L and Rubicon, reciprocally regulate autophagy at different stages. Nat Cell Biol 11: 385–396 19270696 10.1038/ncb1846

[26] Zhong Y , Wang QJ , Li X , Yan Y , Backer JM , Chait BT , Heintz N , Yue Z (2009) Distinct regulation of autophagic activity by Atg14L and Rubicon associated with Beclin 1‐phosphatidylinositol‐3‐kinase complex. Nat Cell Biol 11: 468–476 19270693 10.1038/ncb1854 PMC2664389

[27] Diao J , Liu R , Rong Y , Zhao M , Zhang J , Lai Y , Zhou Q , Wilz LM , Li J , Vivona S *et al* (2015) ATG14 promotes membrane tethering and fusion of autophagosomes to endolysosomes. Nature 520: 563–566 25686604 10.1038/nature14147 PMC4442024

[28] Itakura E , Kishi‐Itakura C , Mizushima N (2012) The hairpin‐type tail‐anchored SNARE syntaxin 17 targets to autophagosomes for fusion with endosomes/lysosomes. Cell 151: 1256–1269 23217709 10.1016/j.cell.2012.11.001

[29] Miyakawa K , Nishi M , Matsunaga S , Okayama A , Anraku M , Kudoh A , Hirano H , Kimura H , Morikawa Y , Yamamoto N *et al* (2017) The tumour suppressor APC promotes HIV‐1 assembly via interaction with Gag precursor protein. Nat Commun 8: 14259 28134256 10.1038/ncomms14259 PMC5290283

[30] Mei Y , Su M , Sanishvili R , Chakravarthy S , Colbert CL , Sinha SC (2016) Identification of BECN1 and ATG14 coiled‐coil interface residues that are important for starvation‐induced autophagy. Biochemistry 55: 4239–4253 27383850 10.1021/acs.biochem.6b00246 PMC5116031

[31] Zhang T , Dong K , Liang W , Xu D , Xia H , Geng J , Najafov A , Liu M , Li Y , Han X *et al* (2015) G‐protein‐coupled receptors regulate autophagy by ZBTB16‐mediated ubiquitination and proteasomal degradation of Atg14L. Elife 4: e06734 25821988 10.7554/eLife.06734 PMC4421748

[32] Ricci V (2016) Relationship between VacA toxin and host cell autophagy in *Helicobacter pylori* infection of the human stomach: a few answers, many questions. Toxins (Basel) 8: E203 27376331 10.3390/toxins8070203 PMC4963836

[33] Corona AK , Saulsbery HM , Corona Velazquez AF , Jackson WT (2018) Enteroviruses remodel autophagic trafficking through regulation of host SNARE proteins to promote virus replication and cell exit. Cell Rep 22: 3304–3314 29562185 10.1016/j.celrep.2018.03.003 PMC5894509

[34] Mohamud Y , Shi J , Qu J , Poon T , Xue YC , Deng H , Zhang J , Luo H (2018) Enteroviral infection inhibits autophagic flux via disruption of the SNARE complex to enhance viral replication. Cell Rep 22: 3292–3303 29562184 10.1016/j.celrep.2018.02.090

[35] Ding B , Zhang G , Yang X , Zhang S , Chen L , Yan Q , Xu M , Banerjee AK , Chen M (2014) Phosphoprotein of human parainfluenza virus type 3 blocks autophagosome‐lysosome fusion to increase virus production. Cell Host Microbe 15: 564–577 24832451 10.1016/j.chom.2014.04.004

[36] Ogawa M , Suzuki T , Tatsuno I , Abe H , Sasakawa C (2003) IcsB, secreted via the type III secretion system, is chaperoned by IpgA and required at the post‐invasion stage of *Shigella* pathogenicity. Mol Microbiol 48: 913–931 12753186 10.1046/j.1365-2958.2003.03489.x

[37] Ogawa M , Yoshikawa Y , Kobayashi T , Mimuro H , Fukumatsu M , Kiga K , Piao Z , Ashida H , Yoshida M , Kakuta S *et al* (2011) A Tecpr1‐dependent selective autophagy pathway targets bacterial pathogens. Cell Host Microbe 9: 376–389 21575909 10.1016/j.chom.2011.04.010

[38] Katoh Y , Nozaki S , Hartanto D , Miyano R , Nakayama K (2015) Architectures of multisubunit complexes revealed by a visible immunoprecipitation assay using fluorescent fusion proteins. J Cell Sci 128: 2351–2362 25964651 10.1242/jcs.168740

[39] Kuma A , Hatano M , Matsui M , Yamamoto A , Nakaya H , Yoshimori T , Ohsumi Y , Tokuhisa T , Mizushima N (2004) The role of autophagy during the early neonatal starvation period. Nature 432: 1032–1036 15525940 10.1038/nature03029

[40] Komatsu M , Waguri S , Koike M , Sou YS , Ueno T , Hara T , Mizushima N , Iwata J , Ezaki J , Murata S *et al* (2007) Homeostatic levels of p62 control cytoplasmic inclusion body formation in autophagy‐deficient mice. Cell 131: 1149–1163 18083104 10.1016/j.cell.2007.10.035

[41] Yamamoto S , Izumiya H , Morita M , Arakawa E , Watanabe H (2009) Application of lambda red recombination system to *Vibrio cholerae* genetics: simple methods for inactivation and modification of chromosomal genes. Gene 438: 57–64 19268696 10.1016/j.gene.2009.02.015

[42] Okura M , Osaki M , Fittipaldi N , Gottschalk M , Sekizaki T , Takamatsu D (2011) The minor pilin subunit Sgp2 is necessary for assembly of the pilus encoded by the *srtG* cluster of *Streptococcus suis* . J Bacteriol 193: 822–831 21148736 10.1128/JB.01555-09 PMC3028668

[43] Takai K , Endo Y (2010) The cell‐free protein synthesis system from wheat germ. Methods Mol Biol 607: 23–30 20204845 10.1007/978-1-60327-331-2_3

[44] Zhang JR , Mostov KE , Lamm ME , Nanno M , Shimida S , Ohwaki M , Tuomanen E (2000) The polymeric immunoglobulin receptor translocates pneumococci across human nasopharyngeal epithelial cells. Cell 102: 827–837 11030626 10.1016/s0092-8674(00)00071-4

[45] Agarwal V , Hammerschmidt S (2009) Cdc42 and the phosphatidylinositol 3‐kinase‐Akt pathway are essential for PspC‐mediated internalization of pneumococci by respiratory epithelial cells. J Biol Chem 284: 19427–19436 19473971 10.1074/jbc.M109.003442 PMC2740568

[46] Waterhouse A , Bertoni M , Bienert S , Studer G , Tauriello G , Gumienny R , Heer FT , de Beer TAP , Rempfer C , Bordoli L *et al* (2018) SWISS‐MODEL: homology modelling of protein structures and complexes. Nucleic Acids Res 46: W296–W303 29788355 10.1093/nar/gky427 PMC6030848

