# Supplementary material for: Streptococcus pneumoniae hijacks host autophagy by deploying CbpC as a decoy for Atg14 depletion
Source: EMBO Rep. 2020 Apr 2;21(5):e49232. doi: 10.15252/embr.201949232 (PMC7202210; doi:10.15252/embr.201949232)
Supplement: Supplementary file 1 — Appendix [file EMBR-21-e49232-s001.pdf]

Appendix PDF

Table of Contents:

The Appendix PDF contains Appendix Tables S1, S2, S3, and S4:

- Appendix Table S1 page 2
- Appendix Table S2 page 6
- Appendix Table S3 page 7
- Appendix Table S4 page 8

Appendix Table S1. Sequences of expression vector cloning primers.

CbpA-F	ggaagatctatgtttgcatcaaaaagcgaaag
CbpA-R	ggccgacgtcgacttagtttacccattcaccattggc
$CbpC_{T4}$-F	ggggagatctatgaagcttttgaaaaaaat
$CbpC_{T4}$-R	gggggaattcttaaatccactcaccagatg
CbpD-F	ggggagatctatgaaaattttaccgtttat
CbpD-R	gggggaattcctatactcgttctccatcac
CbpE-F	ggggagatctatgaaaaagaaattaactag
CbpE-R	gggggaattcctactgttctgattccgatt
$CbpF_{T4}$-F	ggggagatctatgaagcttttgaaaaaaat
$CbpF_{T4}$-R	gggggaattcttacttaacccattcaccat
CbpG-F	ggaagatctatggttttatctaagtattatggagtagccg
CbpG-R	ggccgacgtcgacttaaatccactcaccagatgaagcg
CbpI-F	ggggagatctatggggatggcagcttttaa
CbpI-R	Gggggaattcctagtttacccattcaccat
CbpJ-F	ggggagatctatgaaaattttgaaaaaaac
CbpJ-R	gggggaattcttaccgaacccattcgccat
CbpL-F	cgcggatccatgaataaacgtctattttcaaaaatgagtctgg
CbpL-R	ccgctcgagttaatcatctaaatgatcaatggcatattgagc
CbpM-F	ggaaggtctatggtaaaaagacgtataaggag
CbpM-R	ggccgacgtcgacttaacgcacccattcaccattatc
$CbpF_{R6}$-F	ggaagatctatgaaacttttgaaaaaaatgatgc
$CbpF_{R6}$-R	ccggaattcttattgaacccattcgccattatag
LytR-F	ggggagatctatggttaaaaaaattattgg

LytR-R	gggggaattcttaattatcttcatcaccaa
$CbpC_{T4}$-R to 150aa	ccggaattcttaattattatcaaaataataagttctcttttcagc
$CbpC_{T4}$-R to 220aa	ccggaattcttaatctgtaagcatcttacctgaagc
$CbpC_{T4}$-F from 152aa	ggggagatctgctgtaaagacaggctgg
Human Atg14-F	ggaagatctatggcgtctcccagtgggaa
Human Atg14-R	ggccgacgtcgacacggtgtccagtgtaagcttt
Human Atg14-R to 70aa	ggccgacgtcgacttagcggccgtcgaagtagacgaaatc
Human Atg14 CCD-F	ggaagatctgaccgggagaggtttatcgacaag
Human Atg14 CCD-R	ggccgacgtcgacttaggtcttttttctaccaggtcacc
Human Atg14 ΔCCD-F	attgacttaagaagtcattatgagcg
Human Atg14 ΔCCD-R	acttcttaagtcaatgcggccgtcgaagtagacgaaatc
$CbpF_{R6}$-F from 28aa	ccggaattcaataccacaggtggccgatttg
$CbpF_{R6}$-F from 72aa	ccggaattcggttggcaatacttagaaattcctgg
$CbpF_{R6}$-F from 116aa	ccggaattcgataaacaagtactagaggc
$CbpF_{R6}$-R to 27aa	ccgctcgagcgcaaataccgtatttgtagctagc
$CbpF_{R6}$-R to 72aa	ccgctcgagaccgacaaccatctctccaatatcacc
$CbpF_{R6}$-R to 116aa	ccgctcgagtgtttgttctagcaaagcacc
$CbpC_{T4}$ Δloop-F	acttaggttccaagtggtactattttggacaagatg
$CbpC_{T4}$ Δloop-R	acttggaacctaagtattgccaaccgacaacc
$CbpF_{R6}$ Δloop-F	cgcggatccaagtggtactattttggacaag
$CbpF_{R6}$ Δloop-R	cgcggatcctaagtattgccaaccgacaacc
$CbpF_{R6}$ Y83A-F	ttggcaatacttagaaattcctggaacaggtgctcgtgataatttattcga
$CbpF_{R6}$ Y83A -R	tcgaataaattatcacgagcacctgttccaggaatttctaagtattgccaa
$CbpF_{R6}$ E95A-F	cgataaccaaccagttaatgcaattggccttcaggaga

CbpF$_{R6}$ E95A-R	tctcctgaaggccaattgcattaactggttggttatcg
CbpC$_{T4}$ -R to 188aa	ccggaattcttacttagcaacttcaccaattgg
CbpC$_{T4}$ -F from 189aa	ggggagatctggttggactcaagattttcatg
CbpC$_{T4}$ Δdp5-F	taaagacaggttggactcaagattttcatg
CbpC$_{T4}$ Δdp5-R	tccaacctgtctttacagcataattattatca
CbpC$_{T4}$ Δdp6-F	ttgctaaggattggcaaaaagtaaacggaaaa
CbpC$_{T4}$ Δdp6-R	gccaatccttagcaacttcaccaattggtagt
CbpC$_{T4}$ Δdp5&dp6-F	taaagacagattggcaaaaagtaaacggaa
CbpC$_{T4}$ Δdp5&dp6-R	gccaatctgtctttacagcataattattatca
PcpC-F	ccggaattcatgaagcttttgaaaaaaatg
PcpC-R	ccgctcgagttacttaacccattcacc
CbpC$_{T4, dp3J}$-F Insert	ggttggcaatacttaccgtttccatctaaaggtag
CbpC$_{T4, dp3J}$-R Insert	aaaatagtaccactttgactttggaaaaccttctaa
CbpC$_{T4, dp3J}$-F vec	aagtggtactattttggacaag
CbpC$_{T4, dp3J}$-R vec	taagtattgccaaccgacaacc
CbpJ$_{dp3C}$-F Insert	ggctggcaatatatcgaaattcctggaacaggtta
CbpJ$_{dp3C}$-R Insert	gaagtagtaccactcctcctgaaggccaatttca
CbpJ$_{dp3C}$-F vec	gagtggtactacttcgataaaaatg
CbpJ$_{dp3C}$-R vec	gatatattgccagccgacaacc
PcpC$_{dp3C}$-F Insert	ggctggcaatatatagaaattcctggaacaggtta
PcpC$_{dp3C}$-R Insert	aaaataaaaccaatcctcctgaaggccaatttca
PcpC$_{dp3C}$-F vec	gattggttttattttggtcaag
PcpC$_{dp3C}$-R vec	tatatattgccagccgacaacc
Human Beclin1-F	cgcggatccatggaagggtctaagacgtcc

Human Beclin1-R	ggccgacgtcgactcatttgttataaaattgtgaggacaccc
Kozac-HA-Atg14-F	ggaagatctgccgccaccatgtacccatacgatgttccagattacgctatggcgtctcccagtgggaa
Human EGFR-F	cccaagcttgccgccaccatgcgaccctccgggacggc
Human EGFR-R	ccgctcgagtgctccaataaattcactgc
Human UVRAG-F	ggaagatctatgagcgcctccgcgtcgg
Human UVRAG-R	ggccgacgtcgactcacttatcggaactcctgc
Human UVRAG CCD-F	ggaagatctcatagagcccagtgtgcaat
Human UVRAG CCD-R	ggccgacgtcgacttgttgcttatgcagtaatg

Appendix Table S2. Design of the siRNA for knockdown and primer sequences for RT-PCR.

si p62	acaucgauaucaacuucaaugcc
si Atg5	tattctaaaggggatataacgaa
si pIgR	aagctacaggacagacattag
si Luc	aucguacgcggaauacuucgadtdt
check for human p62 KD-F	cagcttctggtccatcggag
check for human p62 KD-R	ggactccaaggcgatcttcc
check for human Atg5 KD-F	tgggccatcaatcggaaact
check for human Atg5 KD-R	tctgttggctgtgggatgat
check for human pIgR KD-F	ctccaccctggtgcccctggg
check for human pIgR KD-R	cctccttggatgacctttttgc
check for human beta Actin-F	gctcgtcgtcgacaacggct
check for human beta Actin-R	caaacatgatctgggtcatcttctc

Appendix Table S3. Sequences of primers for deletion mutant construction.

$CbpC_{T4}$-N-F	gctcaattgcgtgtagcctc
$CbpC_{T4}$-N-R	cgtacgctagcaattgtacattacaaaagcttcattttatatatcctcc
$CbpF_{R6}$-N-R	cgtacgctagcaattgtacattacaaaagtttcatctatatatcctcc
$CbpC_{T4}$-C-F	aatggactaatgaaaatgtaaatttaactacatctggtgagtggatttaa
$CbpC_{T4}$-C-R	cgggcgtttcggtgcagttagc
$CbpF_{R6}$-C-F	aatggactaatgaaaatgtaaatttaactaggcgaatgggttcaataatg
$CbpF_{R6}$-C-R	gacctcattttgtagctgacc
erm-F	tgtacaattgctagcgtacg
erm-R	tagttaaatttacattttcattagtccatt
delta *cbpc* for check-F	ccgtcaagccctttacttctc
erm mid for check-R	gatctagagctcgaggatcc
CbpA-N-F	cagatgctaccagggtcgtcatg
CbpA-N-R	cgtacgctagcaattgtacattaggcaacagctacactagctactccaatac
CbpA-C-F	aatggactaatgaaaatgtaaatttaactagccaatggtgaatgggtaaac
CbpA-C-R	cgatgagggcgaaaaccctg
delta *cbpA* for check-F	ccaagagtggacagagtgtcg
LytA-N-F	gacaaaagcttgtacgcgga
LytA -N-R	cgtacgctagcaattgtacaccatattctactccttatca
LytA -C-F	aatggactaatgaaaatgtaaatttaactaggcttgattacagtaaaata
LytA -C-R	cttgaagctgtctctataac
delta *lytA* for check-F	aatccgcaggacttgctacc

Appendix Table S4. Sequences of primers for complemented strain construction.

Nt-F2	ctctgtttgattgctgcctg
Nt-R2	gttacacaactgcaactgcttttcactttatcgttatggactggc
Pro-F	aaaagcagttgcagttgtgtaac
CbpC-tag-R	cgtaatctggaacatcgtatgggtattgaacccattcgccattatag
tag-*cm*-F	cccatacgatgttccagattacgcttaatgtacaattgctagcgtacg
cm-R	ttataaaagccagtcattaggcc
Ct-F2	ggcctaatgactggcttttataagctatccaaactatacgagattggggc
Ct-R2	cagctctgtgtcgctctttgcagtcc
Pro-Ext 1	gacatgcagtcgggtctttgctacaatcgttttaggaggatatatagatgaaacttttgaaaaaa atgatgc
Pro-Ext 2	aaaagcagttgcagttgtgtaactgctttttgaggagtttttatatattgacatgcagtcgggtct ttg
Check-F	cctaagtcctcttccataaac
Pro-CbpC F Bglll	ggggagatctaaagtgtttggaagtatga
CbpC-FLAG R6-R Eco	ccggaattcttacttgtcatcgtcatctttgtagtcttgaacccattcgccattatagttg
