# Supplementary material for: Streptococcus pneumoniae hijacks host autophagy by deploying CbpC as a decoy for Atg14 depletion
Source: EMBO Rep. 2020 Apr 2;21(5):e49232. doi: 10.15252/embr.201949232 (PMC7202210; doi:10.15252/embr.201949232)
Supplement: Supplementary file 2 — Expanded View Figures PDF [file EMBR-21-e49232-s002.pdf]

Expanded View Figures

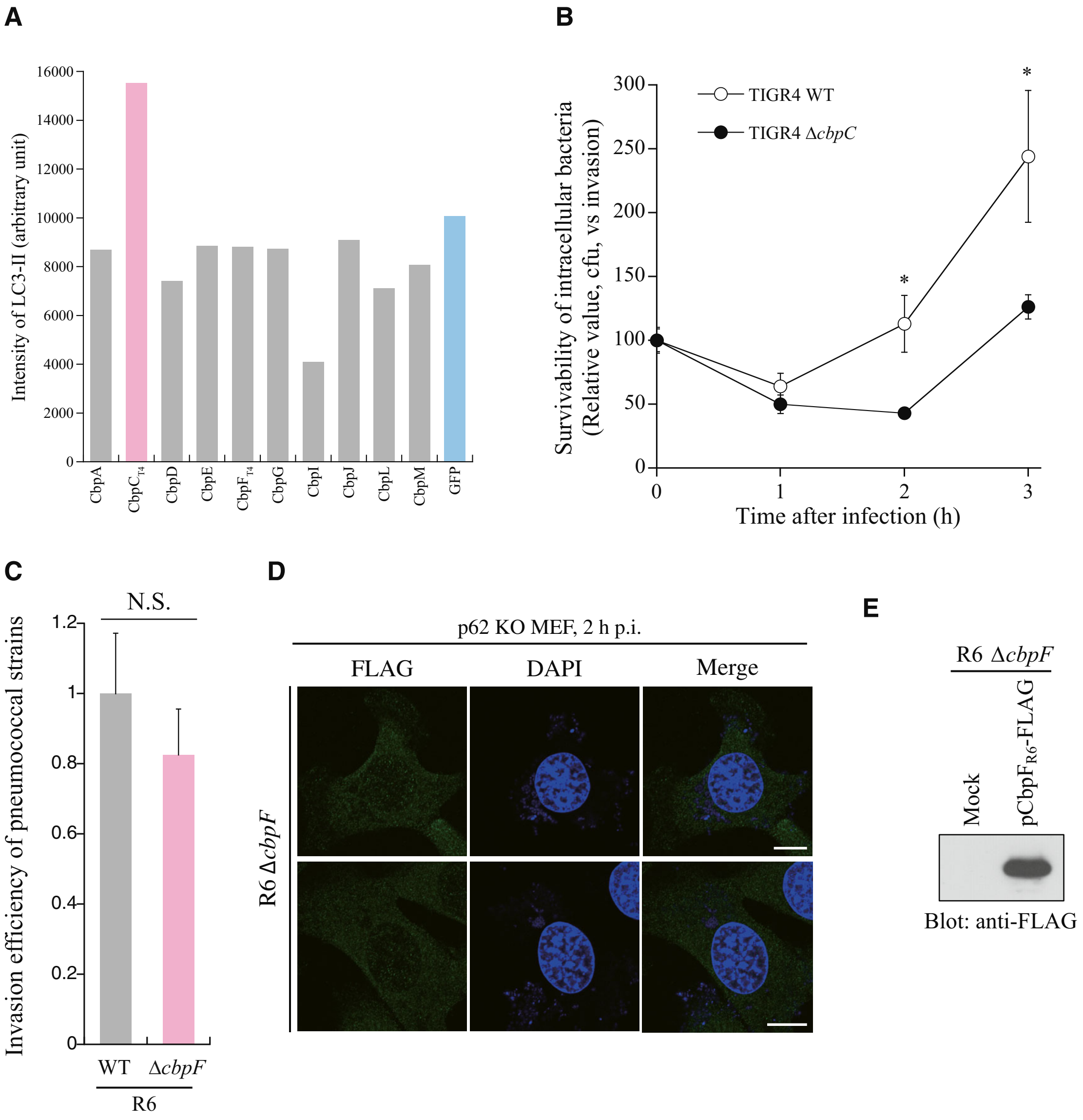

Figure EV1. Pneumococcal CbpC protein can act not only as an autophagy activator but rather as a facilitator for intracellular pneumococcal survivability.

A Quantification of the band intensities shown in Figure 1D.

B MEFs were infected with *S. pneumoniae* TIGR4 WT or Δ*cbpC* for the indicated periods, and intracellular survivability of bacteria was determined as CFU (colony-forming units, $n = 3$).

C MEFs were infected with *S. pneumoniae* R6 WT or Δ*cbpC*, and invasion efficiency of bacteria was determined by CFU.

D p62-KO MEF cells infected with *S. pneumoniae* R6 Δ*cbpF* for 2 h were fixed and stained with DAPI and an anti-FLAG antibody. Representative epifluorescence images are shown. Scale bars, 10 μm.

E Lysates from *S. pneumoniae* R6 Δ*cbpF* or Δ*cbpF* expressing CbpF$_{R6}$-FLAG were subjected to SDS–PAGE and analyzed by immunoblotting using an anti-FLAG antibody.

Data information: In (B, C), data represent mean ± SEM of 3 biological replicates. Student's *t*-test was used to calculate statistical significance. *$P < 0.01$. N.S., not significant.

Source data are available online for this figure.

© 2020 The Authors

Figure EV2. Screening of autophagy-related proteins that interacted with CbpC and sequence analysis of CbpC homologs in the genomes of the *S. pneumoniae* TIGR4 and D39/R6 strains.

A Streptavidin-pulldown assays using *in vitro*-translated biotinylated $CbpC_{T4}$ and FLAG-tagged autophagy-related proteins. Bound proteins were analyzed by immunoblotting using an anti-FLAG antibody.
B Diagram of $CbpF_{R6}$ and $CbpC_{T4}$.
C Sequence alignment of $CbpF_{R6}$ and $CbpC_{T4}$. The identical amino acid residues are indicated by asterisk, and conservative changes are shown by dots.
D GST-pulldown assays using GST-$CbpF_{R6}$ or GST and lysates from 293T expressing the indicated GFP-fused proteins or GFP. Bound proteins were analyzed by immunoblotting using an anti-GFP antibody.
E Phylogenetic tree and schematic representations of CbpC homologs.

Source data are available online for this figure.

© 2020 The Authors

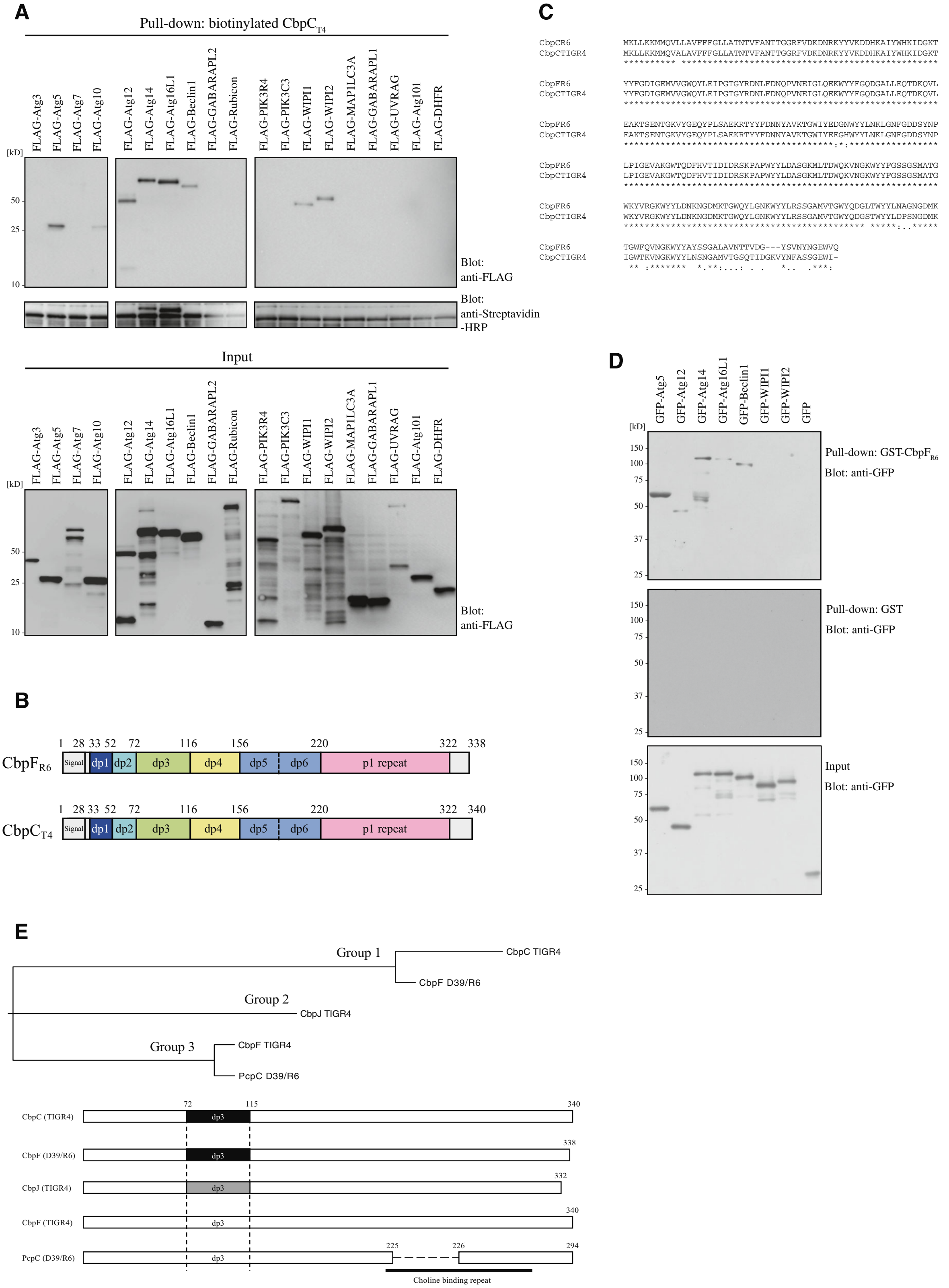

Figure EV2.

 © 2020 The Authors

Figure EV3. CbpC can bind to p62 and act as a decoy for autophagic degradation of Atg14 to suppress autophagic degradation.

A MEFs were infected with *S. pneumoniae* R6 WT or Δ*cbpF*, and the number of internalized bacteria per cell was determined.
B Atg5-KO MEFs were infected with *S. pneumoniae* R6 WT or Δ*cbpF* for the indicated periods, and the intracellular survival of bacteria was determined and expressed as CFUs.
C A549 cells infected with the indicated *S. pneumoniae* strains for 2 h were fixed and stained with DAPI and an anti-Atg14 antibody, and the percentages of perinuclear-localizing Atg14 containing cells were quantified.
D Representative epifluorescence images of the data presented in (C) are shown. Scale bars, 10 μm
E Knockdown effects of the indicated siRNAs were evaluated by RT–PCR and visualized by agarose gel electrophoresis.
F Lysates from 293T cells transiently expressing the indicated proteins were subjected to SDS–PAGE and analyzed by immunoblotting using the indicated antibodies.
G Quantification of NanoBRET signals in 293A cells transiently expressing Nanoluc-Beclin1 and HaloTag-Atg14 in the presence or absence of GFP-CbpC or GFP.
H Lysates from 293T cells transiently expressing the indicated proteins were subjected to IP assays using anti-HA beads, and bound proteins were analyzed by Western blotting using the indicated antibodies.
I Schematic diagram of p62–CbpC–Atg14-driven autophagy subversion in pneumococcal infection.

Data information: In (A, B, C, G), data represent mean ± SEM of 3 biological replicates. Student's *t*-test was used to calculate statistical significance. *$P < 0.01$, N.S., not significant.

Source data are available online for this figure.

© 2020 The Authors

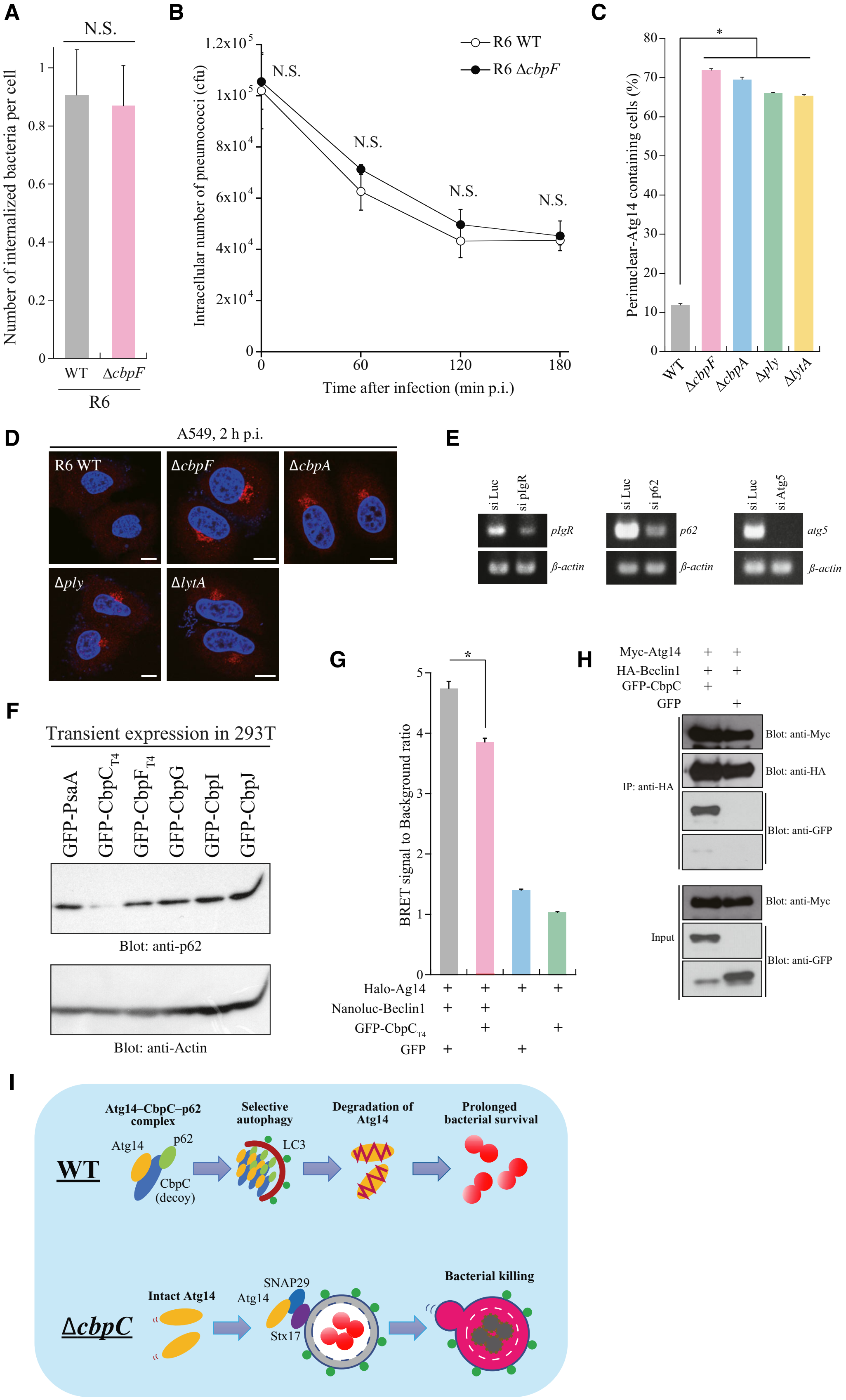

Figure EV3.

 © 2020 The Authors

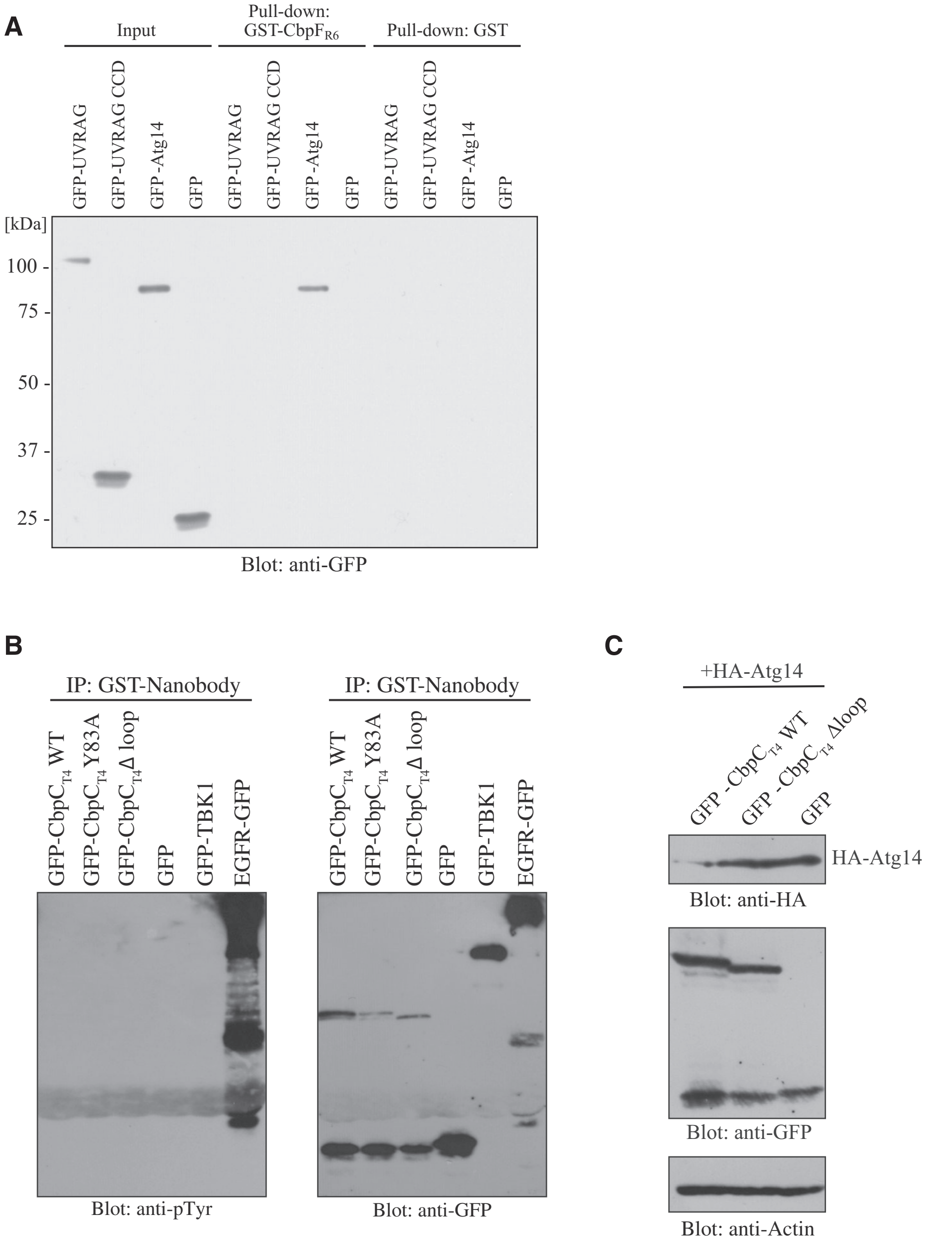

Figure EV4. The loop structure in CbpC dp3 domain interacts with the CCD of Atg14.

A GST-pulldown assays using GST-CbpF$_{R6}$ or GST and lysates from 293T expressing the indicated GFP-fused proteins or GFP were performed. Bound proteins were analyzed by immunoblotting using an anti-GFP antibody.

B Lysates from 293T cells transiently expressing the indicated GFP-fused proteins or GFP in the presence of phosphatase inhibitor were immunoprecipitated with GST-GFP-Nanobody and bound proteins analyzed by immunoblotting using antibodies against phosphotyrosine or GFP.

C Lysates from 293T cells transiently expressing HA-Atg14, and GFP-CbpC, CbpC Δloop, and GFP were subjected to SDS–PAGE and analyzed by immunoblotting with antibodies against HA, GFP, or actin.

Source data are available online for this figure.

Figure EV5. Domain analysis of CbpC–p62 interaction and comparison of Atg14-binding capacity in CbpC family proteins.

A Diagram of $CbpC_{T4}$ derivatives used in (B) and (C).
B Confocal images of Hela cells transiently expressing the indicated $CbpC_{T4}$ derivatives. Scale bars, 10 μm.
C Lysates from 293T cells transiently expressing the indicated $CbpC_{T4}$ derivatives were subjected to SDS–PAGE and then analyzed by immunoblotting using antibodies against LC3B or actin.
D Lysates from 293T cells transiently expressing p62-3Myc and GFP-$CbpC_{T4}$ in the presence or absence of FLAG-TRAF6 (WT or E3 ligase dead) were immunoprecipitated using GST-GFP-Nanobody. Bound proteins were analyzed by immunoblotting.
E Lysates from 293T cells transiently expressing GFP-$CbpC_{T4}$ or GFP and p62-3Myc and FLAG-TRAF6 were immunoprecipitated using GST-GFP-Nanobody. Additionally, beads were mixed with lysates from 293T cells transiently expressing HA-Atg14, and bound proteins were analyzed by immunoblotting.
F Lysates from 293T cells transiently expressing p62-3Myc, GFP-$CbpC_{T4}$ variants, and FLAG-TRAF6 were immunoprecipitated using GST-GFP-Nanobody. Bound proteins were analyzed by immunoblotting.
G Lysates from R6 Δ*cbpF* expressing the indicated $CbpF_{R6}$-HA variants were subjected to SDS–PAGE and analyzed by immunoblotting using an anti-HA antibody.
H Each indicated GST-fused protein-bound bead used in Fig 6D, H, I was confirmed by CBB staining.
I GST-pulldown assays using the indicated GST-fused proteins and lysates from 293T expressing GFP-Atg14 CCD or GFP. Bound proteins were analyzed by immunoblotting using an anti-GFP antibody.

Source data are available online for this figure.

© 2020 The Authors

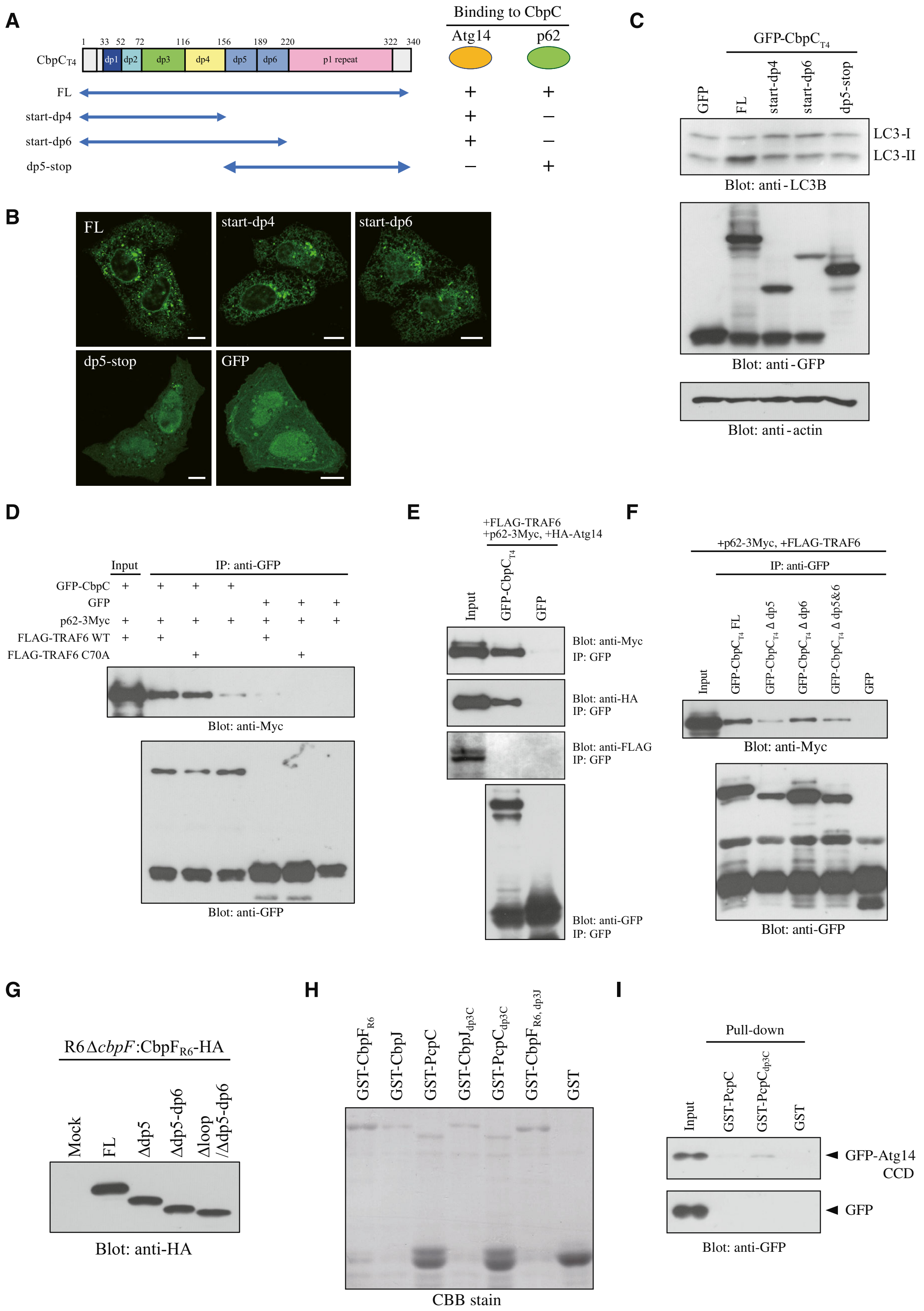
A
Binding to CbpC
Atg14
p62
CbpCT4
1 33 52 72 116 156 189 220 322 340
dp1 dp2 dp3 dp4 dp5 dp6 p1 repeat
FL + +
start-dp4 + −
start-dp6 + −
dp5-stop − +
B
FL
start-dp4
start-dp6
dp5-stop
GFP
C
GFP-CbpCT4
GFP
FL
start-dp4
start-dp6
dp5-stop
LC3-I
LC3-II
Blot: anti-LC3B
Blot: anti-GFP
Blot: anti-actin
D
Input
IP: anti-GFP
GFP-CbpC
GFP
p62-3Myc
FLAG-TRAF6 WT
FLAG-TRAF6 C70A
Blot: anti-Myc
Blot: anti-GFP
E
+FLAG-TRAF6
+p62-3Myc, +HA-Atg14
Input
GFP-CbpCT4
GFP
Blot: anti-Myc
IP: GFP
Blot: anti-HA
IP: GFP
Blot: anti-FLAG
IP: GFP
Blot: anti-GFP
IP: GFP
F
+p62-3Myc, +FLAG-TRAF6
IP: anti-GFP
Input
GFP-CbpCT4 FL
GFP-CbpCT4 Δ dp5
GFP-CbpCT4 Δ dp6
GFP-CbpCT4 Δ dp5&6
GFP
Blot: anti-Myc
Blot: anti-GFP
G
R6ΔcbpF:CbpFR6-HA
Mock
FL
Δdp5
Δdp5-dp6
Δloop /Δdp5-dp6
Blot: anti-HA
H
GST-CbpFR6
GST-CbpJ
GST-PcpC
GST-CbpJdp3C
GST-PcpCdp3C
GST-CbpFR6, dp3J
GST
CBB stain
I
Pull-down
Input
GST-PcpC
GST-PcpCdp3C
GST
GFP-Atg14 CCD
GFP
Blot: anti-GFP

Figure EV5.
