# Supplementary material for: Streptococcus pneumoniae hijacks host autophagy by deploying CbpC as a decoy for Atg14 depletion
Source: EMBO Rep. 2020 Apr 2;21(5):e49232. doi: 10.15252/embr.201949232 (PMC7202210; doi:10.15252/embr.201949232)
Supplement: Supplementary file 3 — Source data for Expanded View [file EMBR-21-e49232-s009.zip › Source_Data_for_EV_Figs/Source_Data_for_FigEV3.pdf]

Fig EV3F

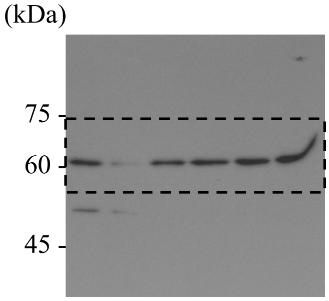

Blot: anti-p62

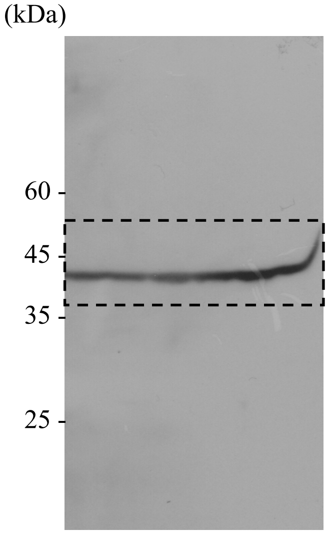

Blot: anti-Actin

Fig EV3H

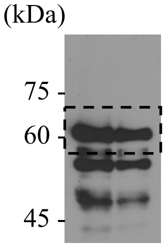

Blot: anti-Myc

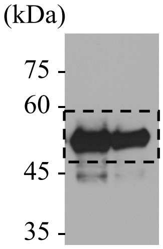

Blot: anti-HA

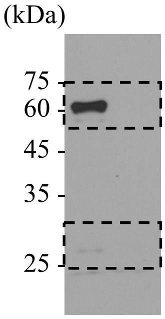

Blot: anti-GFP

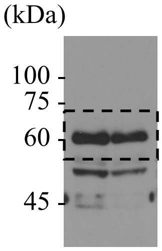

Blot: anti-Myc

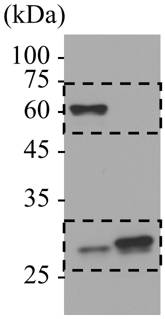

Blot: anti-GFP
