# Supplementary figures and images for: Streptococcus pneumoniae hijacks host autophagy by deploying CbpC as a decoy for Atg14 depletion
Source: EMBO Rep. 2020 Apr 2;21(5):e49232. doi: 10.15252/embr.201949232 (PMC7202210; doi:10.15252/embr.201949232)

**Fig EV1E**

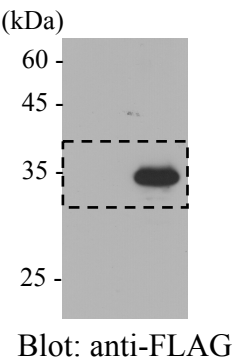

Supplement: Supplementary file 3 — Source data for Expanded View [file EMBR-21-e49232-s009.zip › Source_Data_for_EV_Figs/Source_Data_for_FigEV1.pdf]

**Fig EV2A**

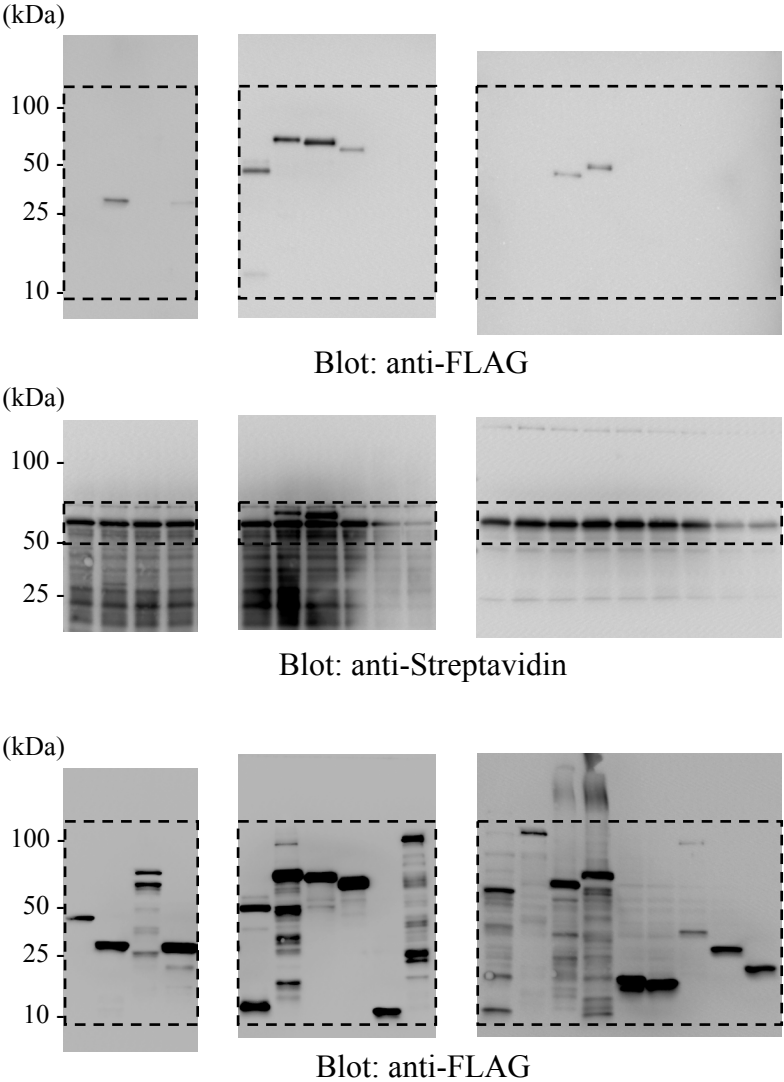

**Fig EV2D**

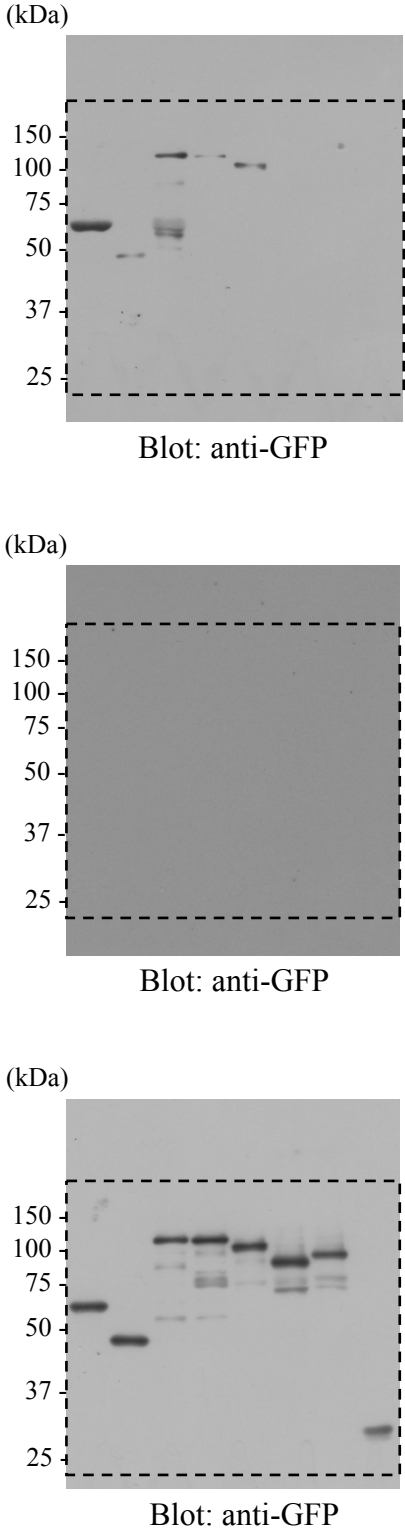

Supplement: Supplementary file 3 — Source data for Expanded View [file EMBR-21-e49232-s009.zip › Source_Data_for_EV_Figs/Source_Data_for_FigEV2.pdf]

**Fig EV4A**

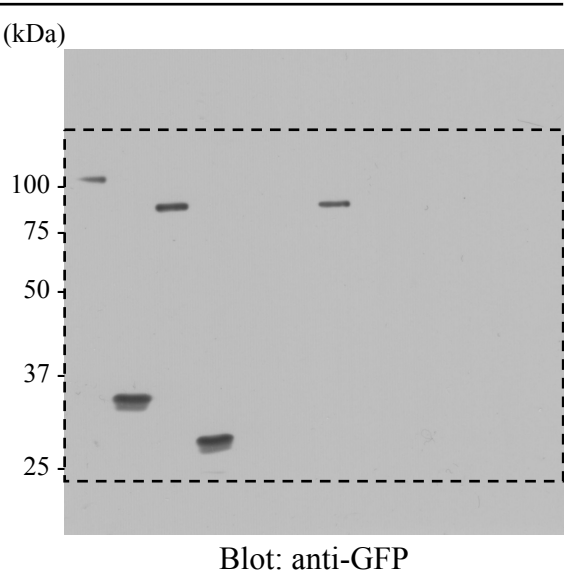

**Fig EV4C**

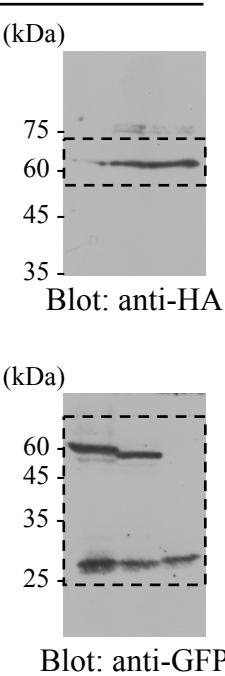

**Fig EV4B**

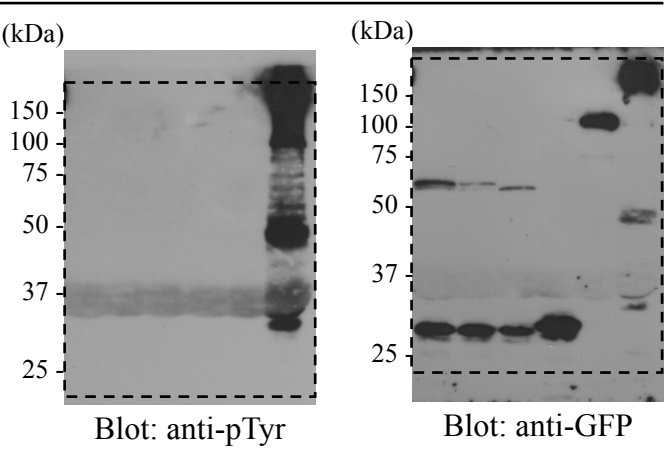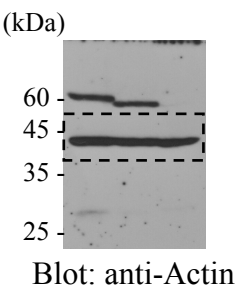

Supplement: Supplementary file 3 — Source data for Expanded View [file EMBR-21-e49232-s009.zip › Source_Data_for_EV_Figs/Source_Data_for_FigEV4.pdf]

**Fig EV5C**

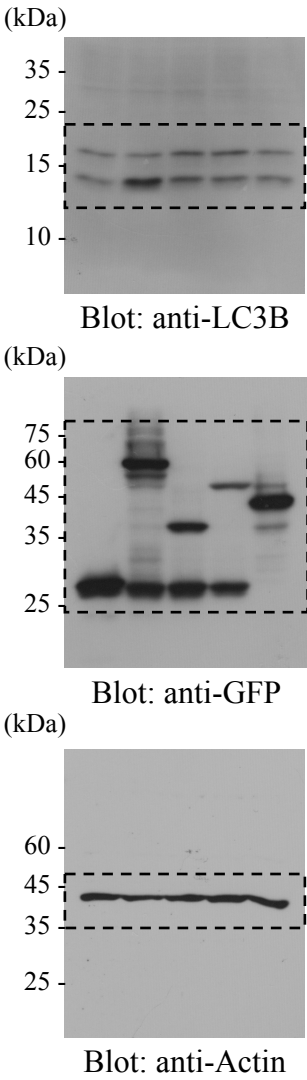

**Fig EV5D**

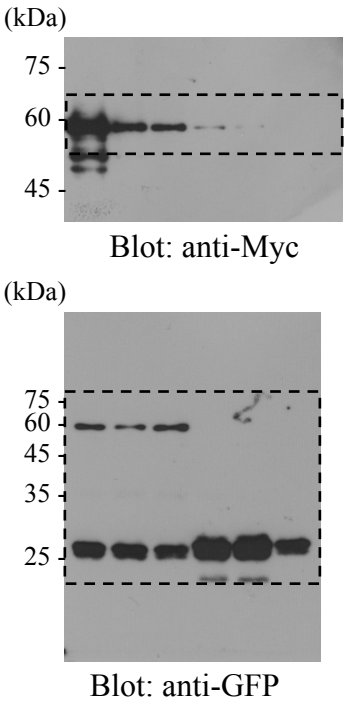

**Fig EV5E**

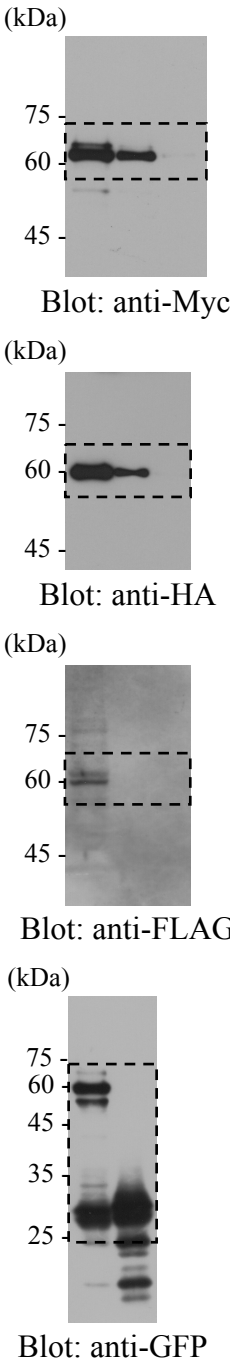

**Fig EV5H**

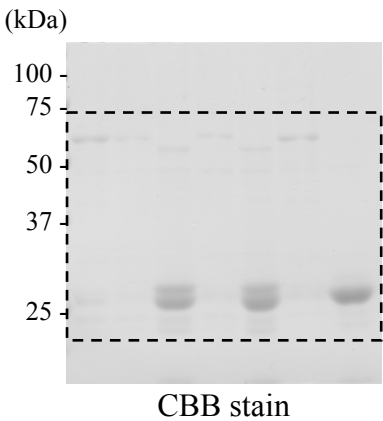

**Fig EV5F**

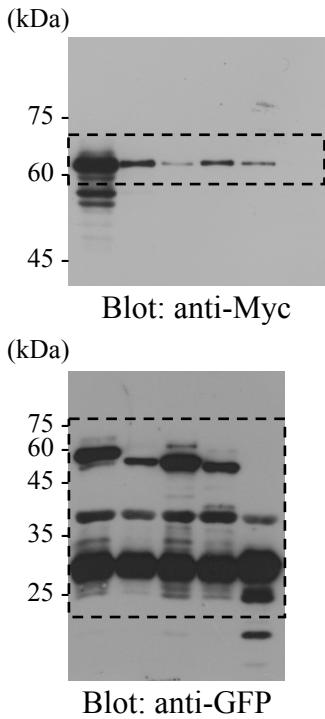

**Fig EV5G**

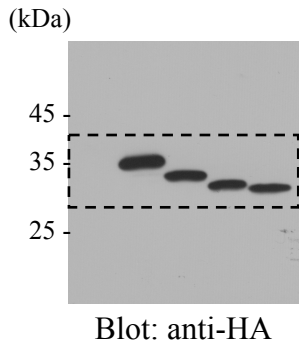

**Fig EV5I**

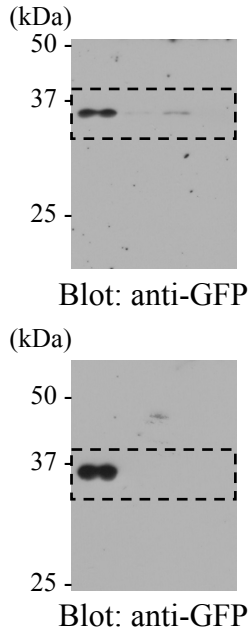

Supplement: Supplementary file 3 — Source data for Expanded View [file EMBR-21-e49232-s009.zip › Source_Data_for_EV_Figs/Source_Data_for_FigEV5.pdf]

**Fig 1D**

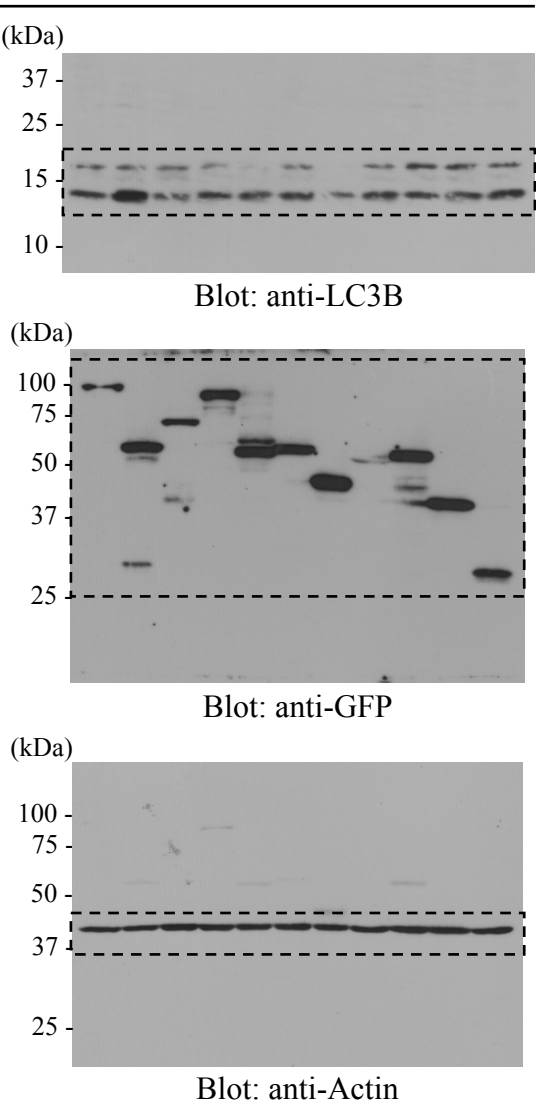

**Fig 1E**

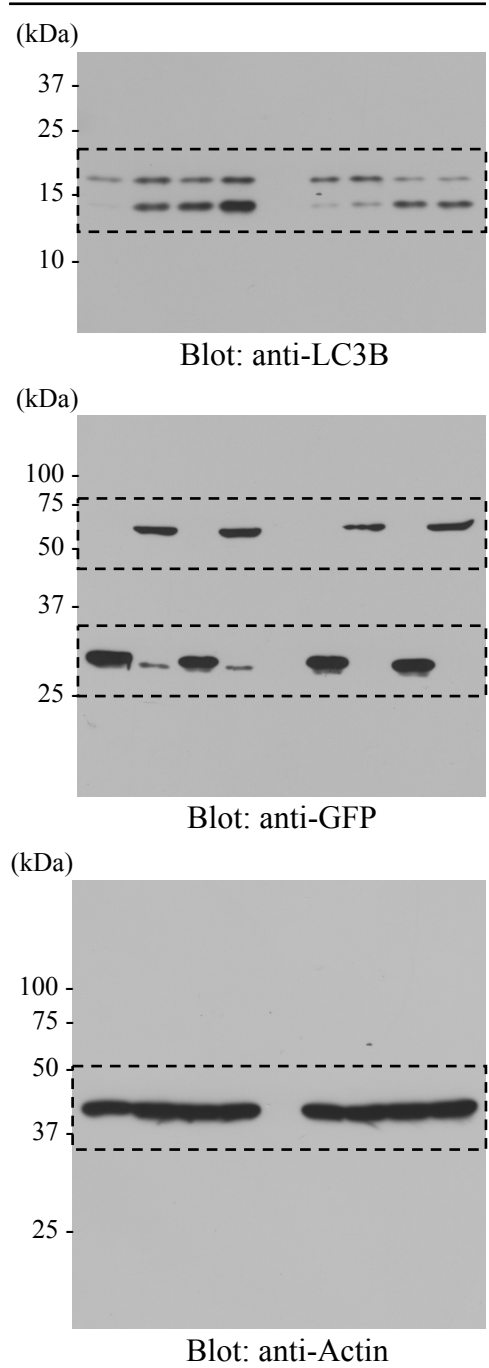

Supplement: Supplementary file 5 — Source data for Figure 1 [file EMBR-21-e49232-s003.pdf]

**Fig 2C**

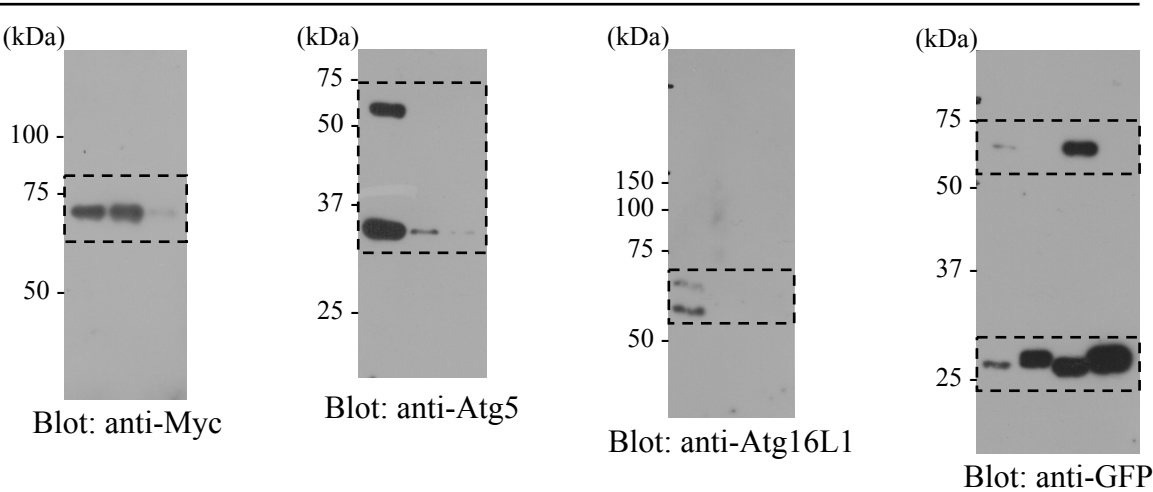

**Fig 2D**

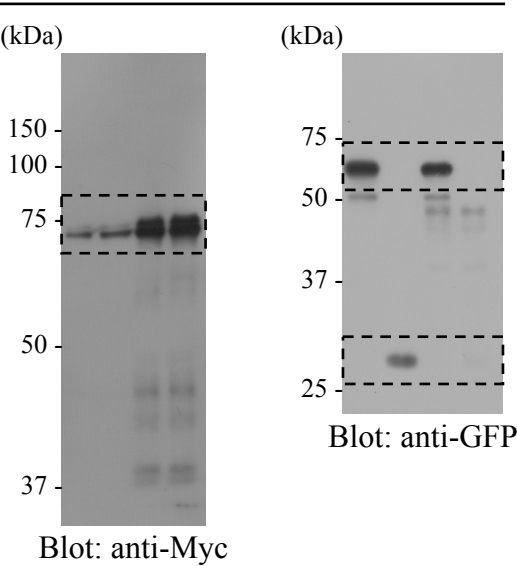

**Fig 2E**

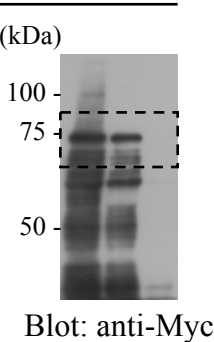

**Fig 2F**

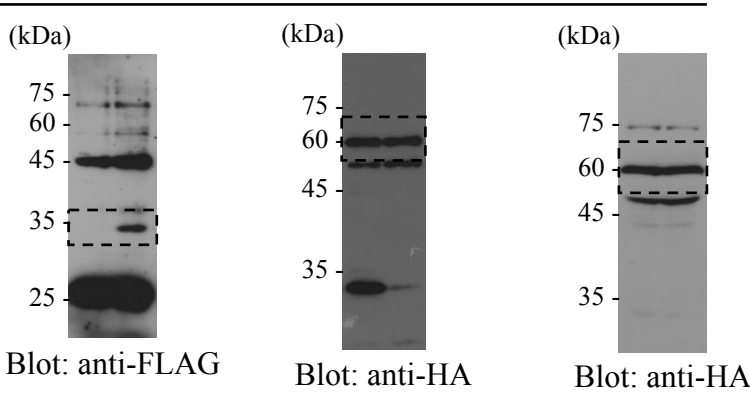

Supplement: Supplementary file 6 — Source data for Figure 2 [file EMBR-21-e49232-s004.pdf]

**Fig 3A**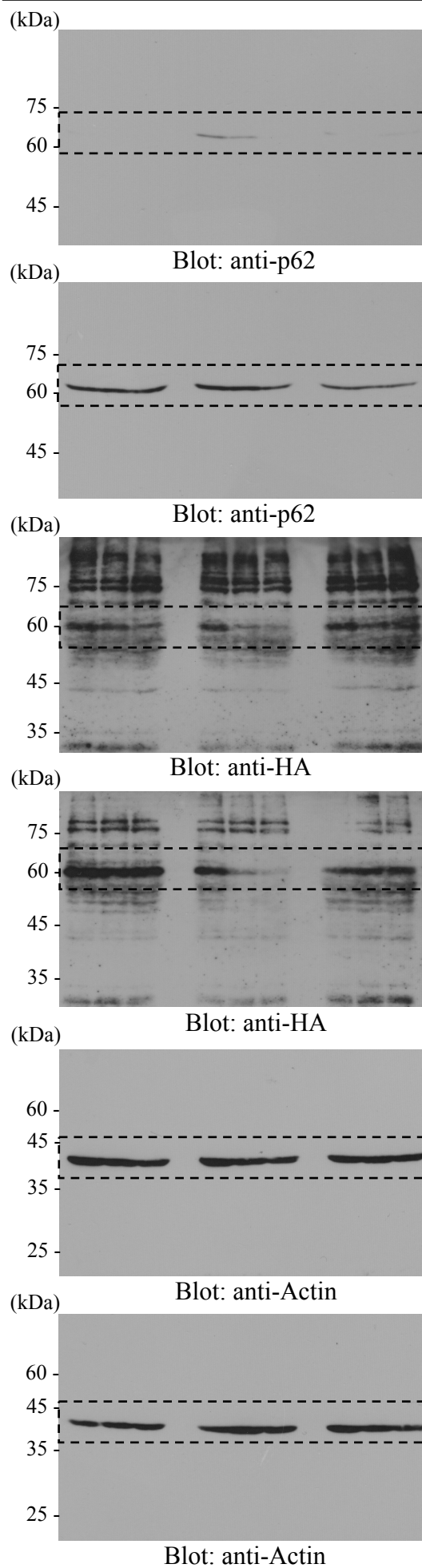**Fig 3B**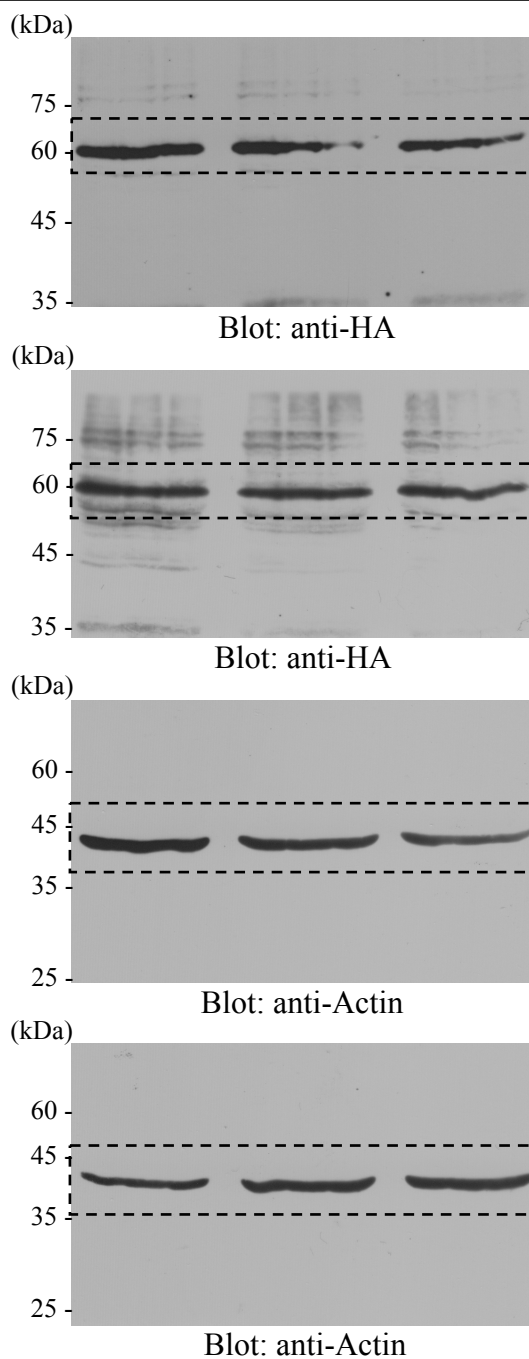**Fig 3L**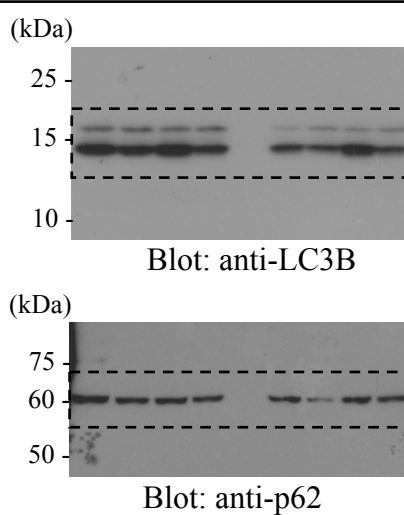**Fig 3I**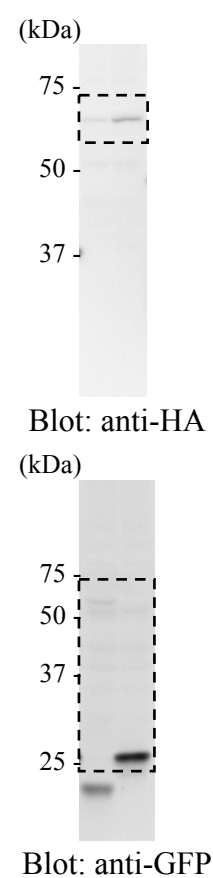**Fig 3J**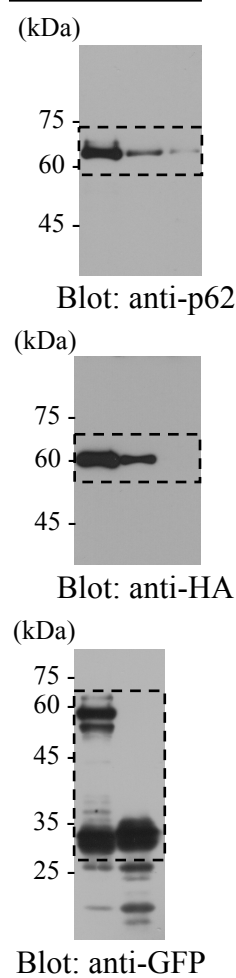

Supplement: Supplementary file 7 — Source data for Figure 3 [file EMBR-21-e49232-s005.pdf]

**Fig 4B**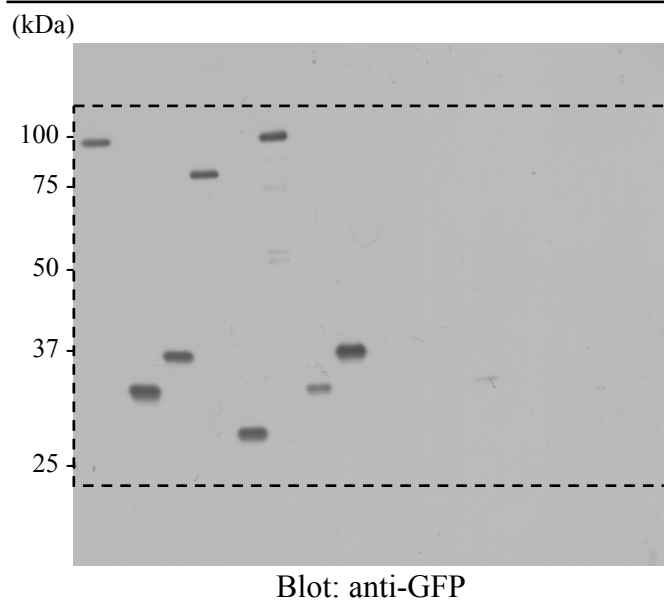**Fig 4E**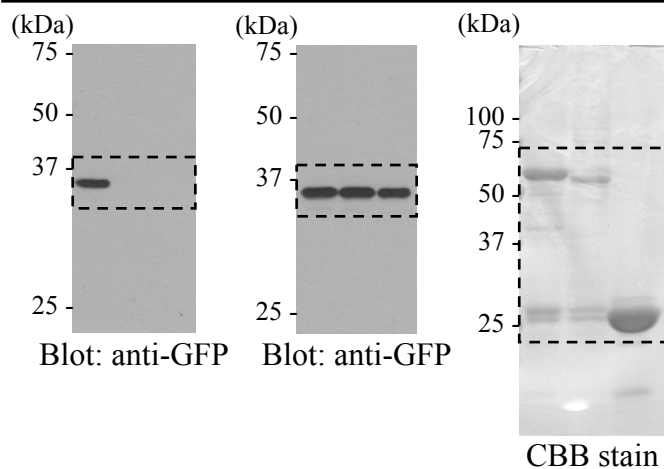**Fig 4D**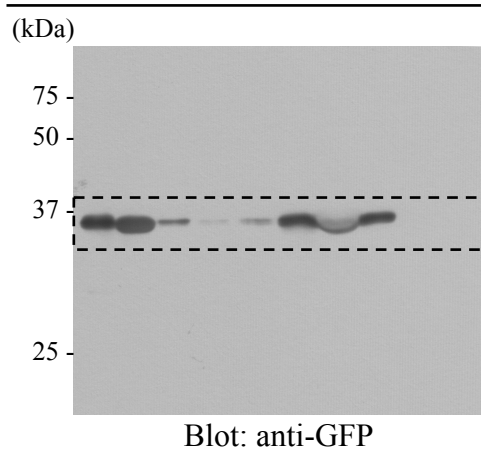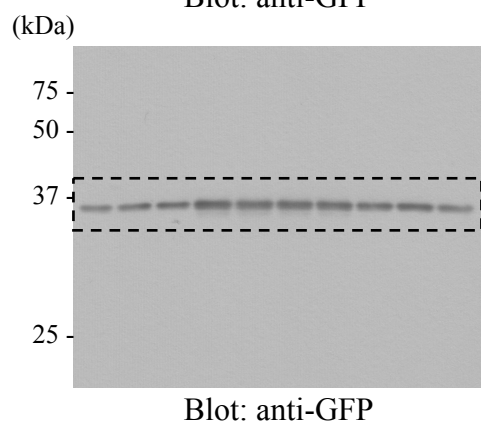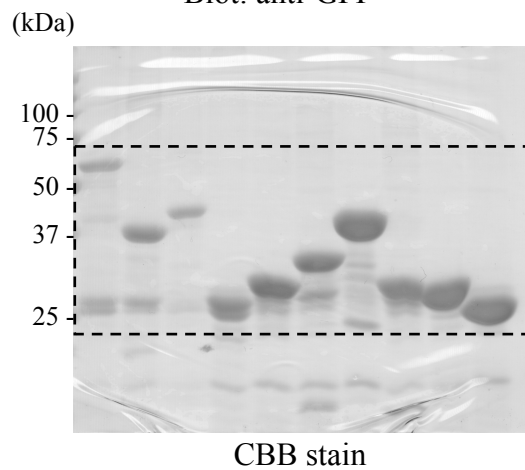**Fig 4H**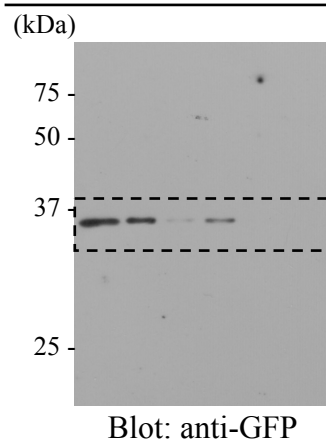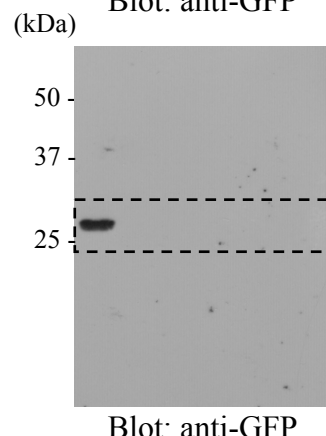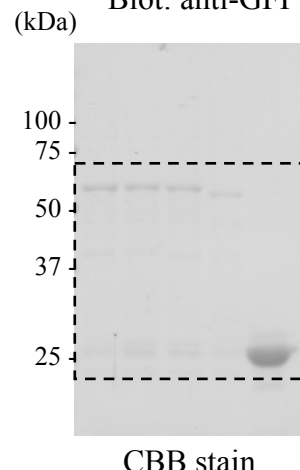**Fig 4I**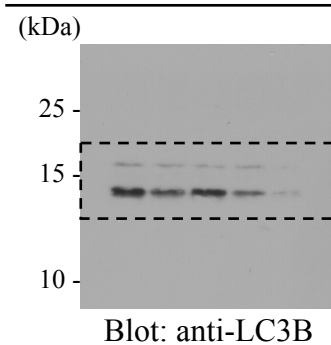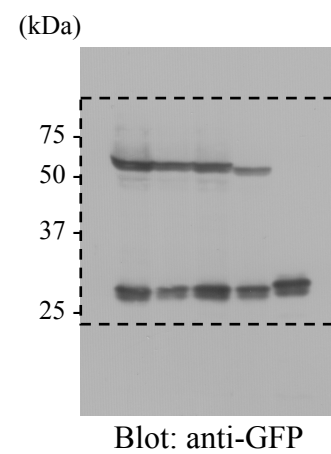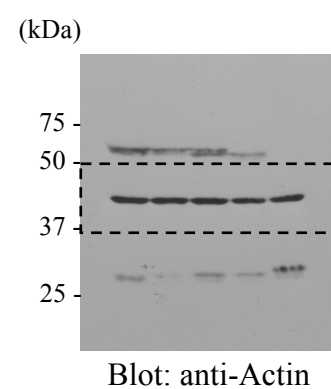

Supplement: Supplementary file 8 — Source data for Figure 4 [file EMBR-21-e49232-s006.pdf]

**Fig 5B**

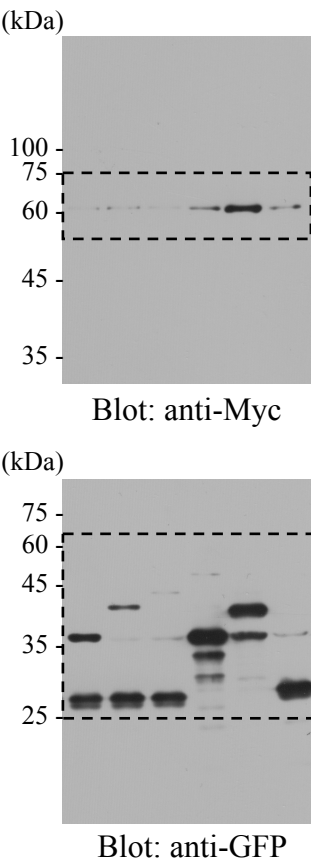

**Fig 5E**

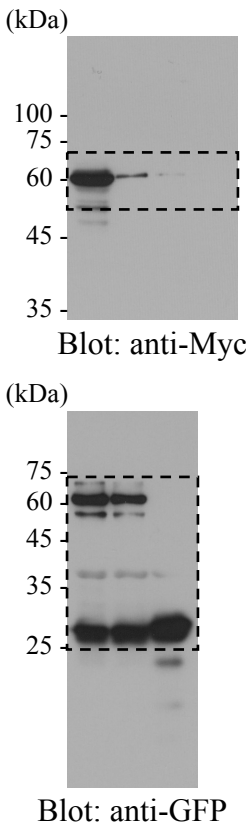

**Fig 5G**

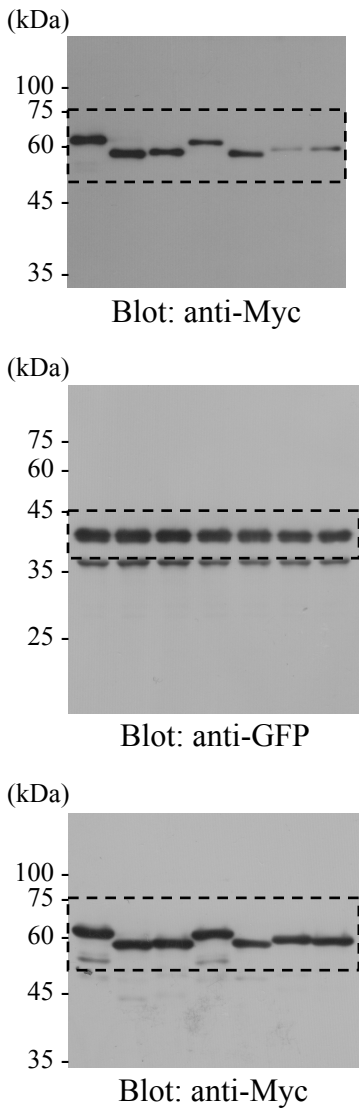

Supplement: Supplementary file 9 — Source data for Figure 5 [file EMBR-21-e49232-s007.pdf]

**Fig 6D**

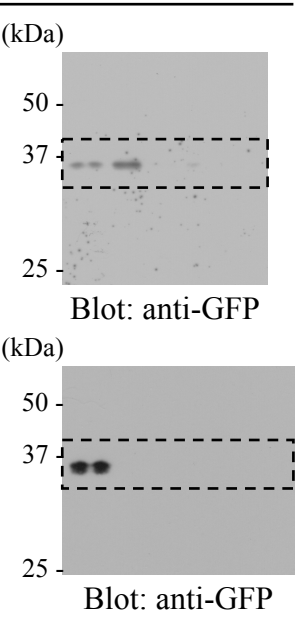

**Fig 6E**

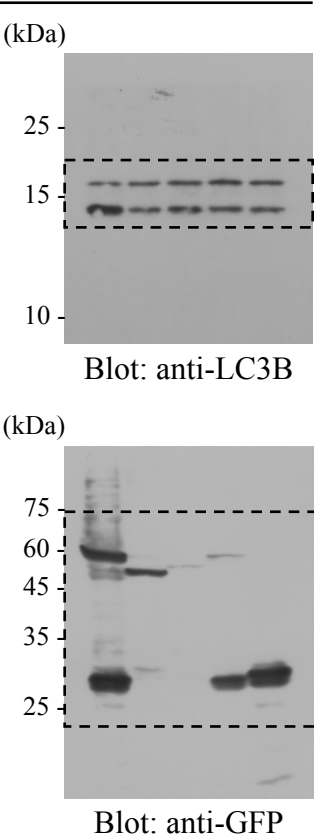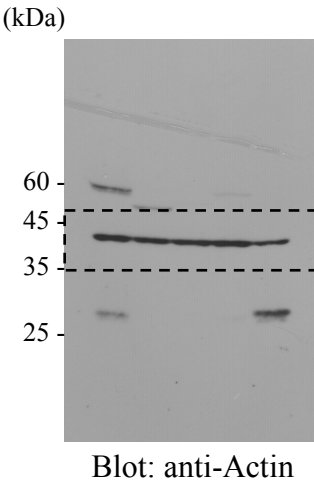

**Fig 6H**

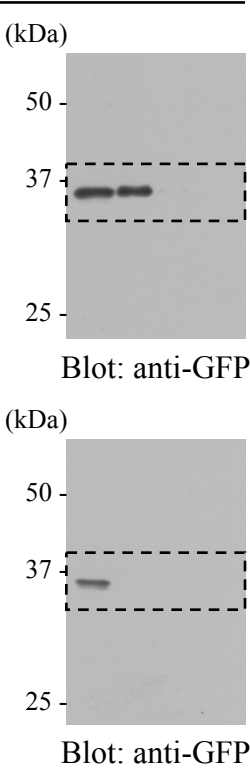

**Fig 6I**

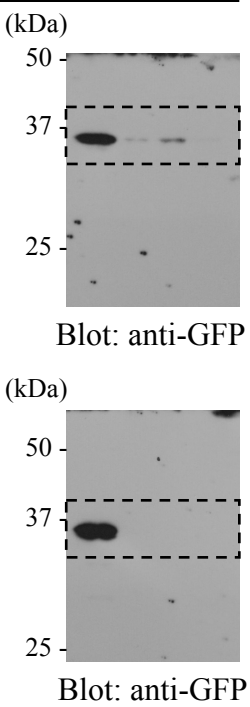

Supplement: Supplementary file 10 — Source data for Figure 6 [file EMBR-21-e49232-s008.pdf]
